# The dynamic etiology and epidemiological patterns of acute respiratory tract infections during and post non-pharmacological interventions of SARS-CoV-2 in Shenzhen, China: a two years’ prospective cohort study from June 2022

**DOI:** 10.3389/fcimb.2025.1599536

**Published:** 2025-09-19

**Authors:** Guohao Fan, Qi Qian, Yimin Tang, Jiexiang Liu, Liuqing Yang, Yun Peng, Yuanlong Lin, Guanyong Ou, Yanling Luo, Chenguang Shen, Yang Yang, Yingxia Liu

**Affiliations:** ^1^ Shenzhen Third People's Hospital, Second Hospital Affiliated to Southern University of Science and Technology, Shenzhen, China; ^2^ Shenzhen Key Laboratory of Pathogen and Immunity, Shenzhen Clinical Research Center for Infectious Disease, State Key Discipline of Infectious Disease, Shenzhen, China; ^3^ Guangdong Key Laboratory for Diagnosis and Treatment of Emerging Infectious Diseases, Shenzhen, China; ^4^ National Clinical Research Center for Infectious Disease, Shenzhen, China; ^5^ Guangdong Medical University, Zhanjiang, China; ^6^ Key Laboratory of Infectious Diseases Research in South China, Southern Medical University, Ministry of Education, Guangzhou, China; ^7^ Department of Laboratory Medicine, Zhujiang Hospital, Guangzhou, China; ^8^ Southern Medical University, Guangzhou, China

**Keywords:** acute respiratory tract infections, etiology, non-pharmacological interventions, prospective cohort study, Southern China

## Abstract

**Background:**

The rigorous non-pharmacological interventions (NPIs) during SARS-CoV-2 outbreak posed a deep impact on the etiology and epidemiology of acute respiratory tract infections (ARTIs). We aimed to elucidate the changing patterns during and post NPIs of SARS-CoV-2 in Shenzhen, China.

**Methods:**

A total of 4610 outpatients with ARTIs from the fever clinic of our hospital were enrolled between June 2022 and May 2024, and nasopharyngeal swabs were collected and tested for twenty-five common respiratory pathogens using well-established RT-qPCR. The two year’s period was further divided into three stages: Stage 1 with strict NPIs, Stage 2 with outbreak of SARS-CoV-2 and Stage 3 with regular epidemic of SARS-CoV-2. Demographic and clinical data were also collected and analyzed.

**Results:**

Overall, 57.05% (2630/4610) of patients were positive for at least one of tested respiratory pathogens, with top five pathogens of IAV (17.09%), *H.influenzae* (13.97%), SARS-CoV-2 (10.11%), IBV (7.38%) and HAdV (5.66%). Except for SARS-CoV-2, IAV and *H.influenzae* dominated the three stages, while the other pathogens varied. Meanwhile, positivity rates of most viral pathogens have increased post NPIs. Moreover, HAdV and *H.influenzae* infections were more frequently found in males. and higher overall rates of viral and bacterial infections were found in both children and the elderly. Notably, the results indicate a higher positivity rate in summer and autumn, with the lowest rate observed in winter. The overall co-infection rate was 24.62%, and the most frequent co-infections were between IAV, SARS-CoV-2, HAdV and *H.influenzae*.

**Conclusions:**

In conclusion, the etiology and epidemiological patterns of ARTIs during and post NPIs of SARS-CoV-2 in Shenzhen have changed overtime, and sex, age and seasonal patterns were also found. The findings could provide useful information for the public health measures and the clinical management of respiratory infections.

## Introduction

Acute respiratory tract infections (ARTIs) are caused by the invasion of the respiratory system by pathogens, including viruses, bacteria, and atypical microorganisms (Claassen-Weitz et al., 2021; Wang et al., 2021). ARTIs pose a serious global health issue with high incidence, rapid transmission, and mortality, resulting in a considerable disease burden annually (GBD 2019 Diseases and Injuries Collaborators, 2020; Li et al., 2021). According to the World Health Organization (WHO), respiratory infections represent the fourth leading cause of mortality, with nearly 3 million deaths globally in 2016 (Mortality and global health estimates). Particularly in low and middle-income countries, ARTI contributes to a greater disease burden. The outbreak of severe acute respiratory syndrome coronavirus 2 (SARS-CoV-2) in 2019 resulted in millions of deaths significantly disrupting societies, and devastated economies, underscored the need for increased attention to ARTIs (COVID-19 cases | WHO COVID-19 dashboard; Li et al., 2020).

The implementation of non-pharmaceutical interventions (NPIs), such as wearing masks and social distancing measures, to reduce the transmission of SARS-CoV-2 has resulted in notable shifts in the spectrum and epidemiology of respiratory infection pathogens (Chow et al., 2023; Park et al., 2023). The typical seasonal cyclic patterns of common respiratory infections have been substantially disrupted under the influence of the pandemic (Chow et al., 2023; Wei et al., 2024). For example, the activity of influenza Virus (IV) and respiratory syncytial virus (RSV) remained remarkably lower during the usual circulating season in multiple countries (Adlhoch et al., 2021; van Summeren et al., 2021; Chow et al., 2023; Zhao et al., 2025). The overall number of twelve notifiable infectious diseases and five non-infectious respiratory diseases in Pakistan declined by 52.3% in 2020 (Rana et al., 2021). A total of 514,341 cases of 39 types of notifiable infectious diseases (NIDs) were reported in Guangdong during the emergency response period in 2020, which decreased by 50.7% compared with the synchronous period during 2015-2019 (Xiao et al., 2021). Following the cessation of NPIs, there was a notable shift in the ARTIs pathogen profile, with an increase in the detection of pathogens such as respiratory syncytial virus (RSV), influenza virus (IFV), human adenovirus (HAdV), and *Mycoplasma pneumoniae* (Wei et al., 2024). As indicated in the January 2022 weekly sentinel surveillance report from New South Wales, Australia, all included respiratory viruses exhibited substantially reduced activity in 2020 compared to the 2015–2019 average, with rhinovirus (RhV) and HAdV showing the least suppression, and RSV remaining suppressed until an off-season resurgence in September 2020. In contrast, parainfluenza virus (PIV) and metapneumovirus (MPV) displayed limited circulation until mid-2021, while IFV circulation persisted at very low levels through the end of 2021 (Pneumonia Etiology Research for Child Health (PERCH), 2021). In America, during the 2020–2021 influenza season, the number of cases plummeted to an all-time low. According to data from the Centers for Disease Control and Prevention (CDC), the outpatient visit rate for influenza-like illness (ILI) was only 0.8%, which is a 94% decrease compared to the average rate during the same period over the previous five years. Following the relaxation of NPIs in spring 2022, however, the prevalence of the influenza A (H3N2) virus rebounded markedly. Seattle surveillance data revealed that the detection rate increased from 0.35% during the lockdown period to 1.15% (2021–2022 season | Influenza (Flu) | CDC; Zhao et al., 2025). Similar asynchronous resurgence was also noted in other countries with varied timing and stridency of NPIs (Kuitunen et al., 2022; Li et al., 2022). Although studies have set out to elucidate these changes (Feng et al., 2014; Li et al., 2021; Snoeck et al., 2021), existing studies have been limited by either a small number of detected pathogens or targeted groups of participants. Moreover, most descriptions have been principally based on cross-sectional studies without longitudinal surveys and analyses which introduced bias from location, climate and demographics.

**Figure 1:**
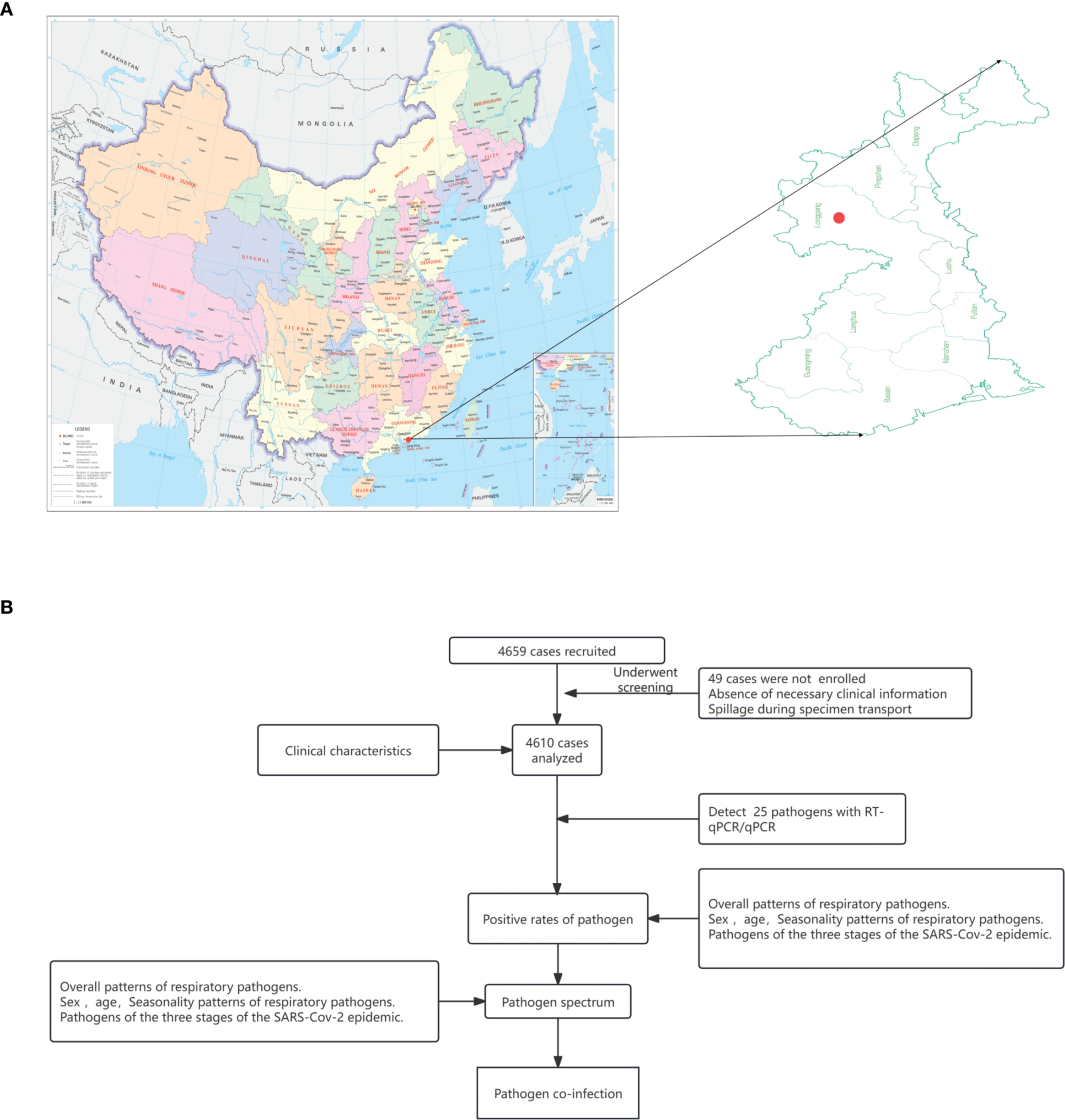
Design of the study. **(A)** The location of Shenzhen, subprovincial city in Guangdong, special economic zone close to Hong Kong and Macao. **(B)** Flow chart of patient recruitment and disposition.

Shenzhen, one of China’s most important port cities close to Hong Kong (**Figure 1A**), faces greater risks of infectious disease importation due to its unique geographical location, population structure, and economic activities. This leads to notable regional characteristics in the respiratory pathogen spectrum which may deeply affect other regions. In this study, we conducted an in-depth longitudinal investigation into the etiology and epidemiological patterns of ARTIs during and post NPIs of SARS-CoV-2 in Shenzhen, China, based on a two years’ prospective cohort study of all ages.

## Materials and methods

### Sample collection

This study focused on patients presenting with febrile respiratory symptoms and included those who sought treatment at the Fever Clinic of Shenzhen Third People’s Hospital from June 2022 to May 2024 (**Figure 1B**). The inclusion criteria were based on the presence of clinical symptoms including fever (axillary temperature ≥37.3 °C), cough, phlegm, nasal congestion, sneezing, runny nose, shortness of breath, difficulty breathing, throat discomfort, dry or sore throat, chest pain, muscle aches, and abnormal breath sounds upon auscultation (e.g., moist rales, dry rales, wheezing, and dullness). All enrolled patients diagnosed with ARTIs provided informed consent, which was signed either by the patients themselves or their family members. Basic patients information (including sex, age and symptoms) and nasopharyngeal swabs were collected. The enrolled patients and collected samples have been divided into three stages for further analyses: Stage 1: Jun to Nov 2022 with NPIs; Stage 2: December 2022 to Jun 2023 with two outbreaks of SARS-CoV-2 after the deregulation of the NPIs; Stage 3: Jul 2023 to May 2024 with regular epidemic of SARS-CoV-2 (Details in the **Supplementary Information**).

### Sample management and laboratory testing

The collected nasopharyngeal swabs were subjected for nucleic acid extraction and the detection of common respiratory pathogens within 72 hours. The detected pathogens include 16 viruses (SARS-CoV-2, influenza A virus (IAV), influenza B virus (IBV), RhV, RSV, AdV, human bocavirus (HBoV), human metapneumovirus (HMPV), human parainfluenza virus 1-4 (HPIV-1-4), seasonal coronaviruses (HCoV-229E, HCoV-OC43, HCoV-NL63, HCoV-HKU1), 6 bacteria (*Haemophilus influenzae, Streptococcus pneumoniae, Staphylococcus aureus, Klebsiella pneumoniae, Pseudomonas aeruginosa, Moraxella catarrhalis), Legionella pneumophila, Mycoplasma pneumoniae* and *Chlamydia pneumoniae* using multiplex quantitative real time polymerase chain reaction (RT-qPCR) (Details in the **Supplementary Information**).

### Statistical analysis

The data were analyzed using SPSS Statistics 26.0, R 4.3.3 and CNSknowall (https://www.cnsknowall.com). The chi-square test or two-tailed Fisher’s exact test and false discovery rate (FDR) correction(Benjamini–Hochberg) were used for group comparisons. The significance level was set at α = 0.05, with *P < 0.05* indicating a statistically significant difference. And we performed an interrupted time-series analysis to evaluate the impact of NPIs. Bacteria, *Legionella pneumophila, M.pneumoniae* and *C.pneumoniae* will be analyzed together within the bacterial group.

## Results

### Baseline characteristics of the cohort

A total of 4,659 febrile patients were initially included, among whom 49 patients were excluded due to incomplete data recording or sample quality issues (**Figure 1B**). Consequently, the final analysis encompassed a total of 4610 patients, of which 2371 were male (51.43%) and 2239 were female (48.57%). The median age was 28 years old. Among the patients, there were 1203 individuals with <18 years of age (children group, 26.10%), 3,241 individuals with 18–59 years of age (adult group, 70.30%), and 166 individuals with ≥ 60 years of age (elderly group, 3.60%). As to the time-points of enrollment, 546 (11.84%), 745 (16.16%) and 3319 (72.00%) participants were from Stage 1, 2 and 3, respectively. The average time from symptoms onset to seeking medical attention was 2 days (**Supplementary Figure 1A**). Fever was found in 81.77% of the enrolled patients with 19.15% participants presenting with high fever (≥ 39.0 °C), and significantly higher proportion of fever and high fever were found in viral infections and mixed infections than in bacterial infections (*P < 0.001*). The main symptoms reported included cough (76.83%), runny nose (48.59%), muscle pain (51.69%), and sore throat (50.02%). Nasal congestion, runny nose, dizziness, headache, sore throat, chills fatigue and muscle ache were higher in viral infections than in bacterial infections (*P < 0.05*), while on the contrary for the abdominal pain and diarrhea (**Supplementary Figure 1B**). Notably, the children group showed a higher proportion fever, while higher incidences of other symptoms such as cough, nasal congestion and muscle aches and pains were found in the adult and elderly groups (**Supplementary Figures 1C, D**).

### Overall etiology and epidemiology of the detected respiratory pathogens

**Figure 2:**
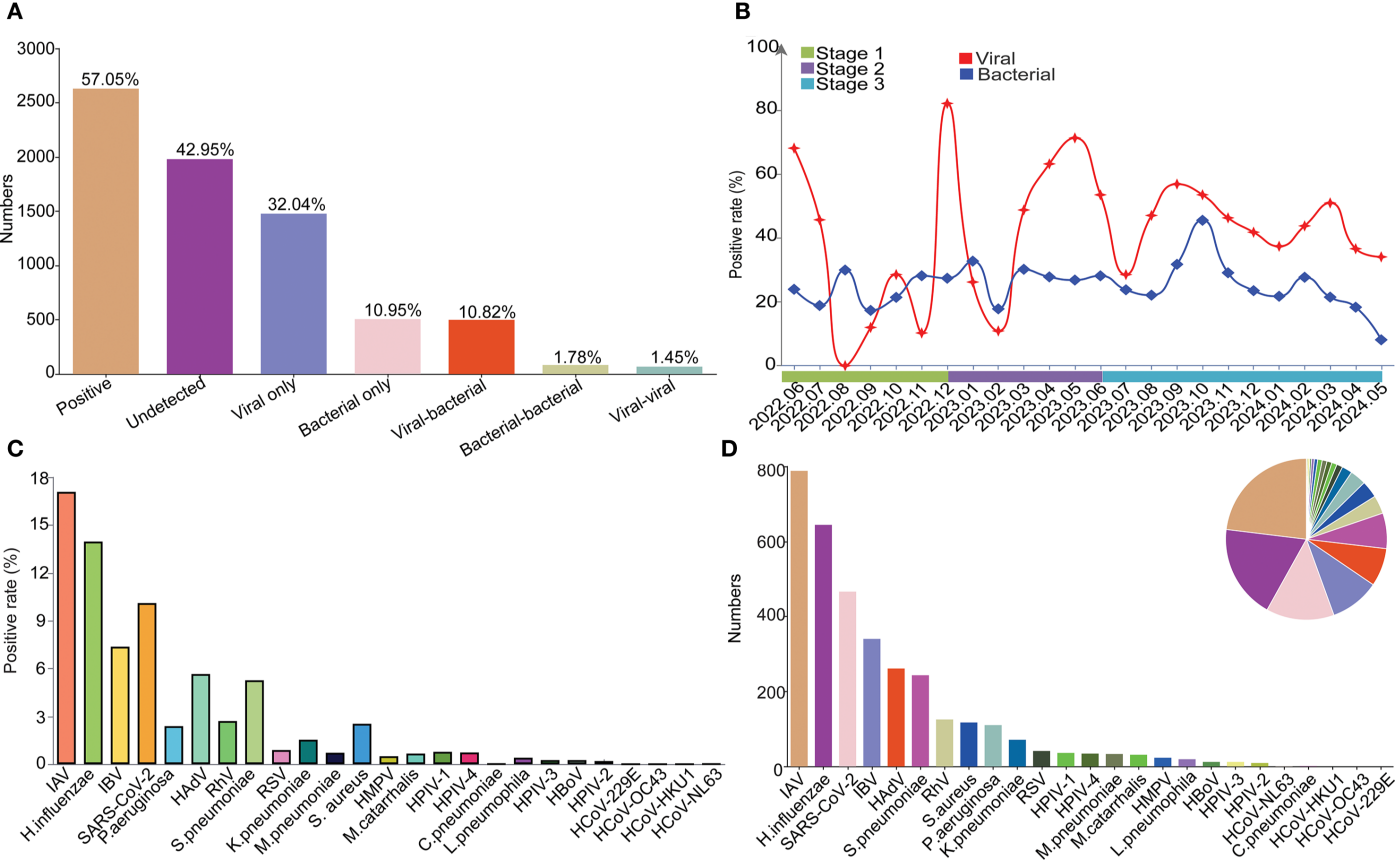
The overall etiology and epidemiology of the detected respiratory pathogens. **(A)** The overall positive rate and infection types among all patients. **(B)** Monthly trends for bacterial and viral infections. **(C)** Positive rates of each pathogen. **(D)** Positive number of each pathogen.

Totally, 57.05% (2630/4610) of the samples were tested positive for at least one pathogen, with 32.04% (1477/4610) and 10.95% (505/4610) for the single infection of virus and bacteria, respectively. Moreover, co-infection between viruses, co-infection between bacteria, and co-infection between virus and bacteria were found in 1.45% (67/4610), 1.78% (82/4610) and 10.82% (499/4610) samples, respectively (**Figure 2A**). Positivity rates of bacteria remained relatively stable around 20%, while the positivity rate of virus fluctuated significantly (**Figure 2B**) with peaks in Jun 2022, Oct 2022, Dec 2022, May 2023, Sep 2023 and Mar 2024. Specifically, the highest positivity rate was IAV with 17.09% (788/4610), followed by *H.influenzae* (13.97%, 644/4610), SARS-CoV-2 (10.11%, 466/4610), IBV (7.38%, 343/4610), HAdV (5.66%, 261/4610), *S.pneumoniae* (5.27%, 243/4610), RhV (2.71%, 125/4610), *S. aureus* (2.54%, 117/4610), *P.aeruginosa* (2.39%, 110/4610), *K.pneumoniae* (1.54%, 71/4610) within the top 10 pathogens. For the 4 seasonal coronaviruses of HCoV-229E, HCoV-NL63, HCoV-OC43 and HCoV-HKU1, only one sample was tested positive for HCoV-NL63 (**Figures 2C, D**).

### Comparative etiology and epidemiological patterns during and post NPIs of SARS-CoV-2

**Figure 3:**
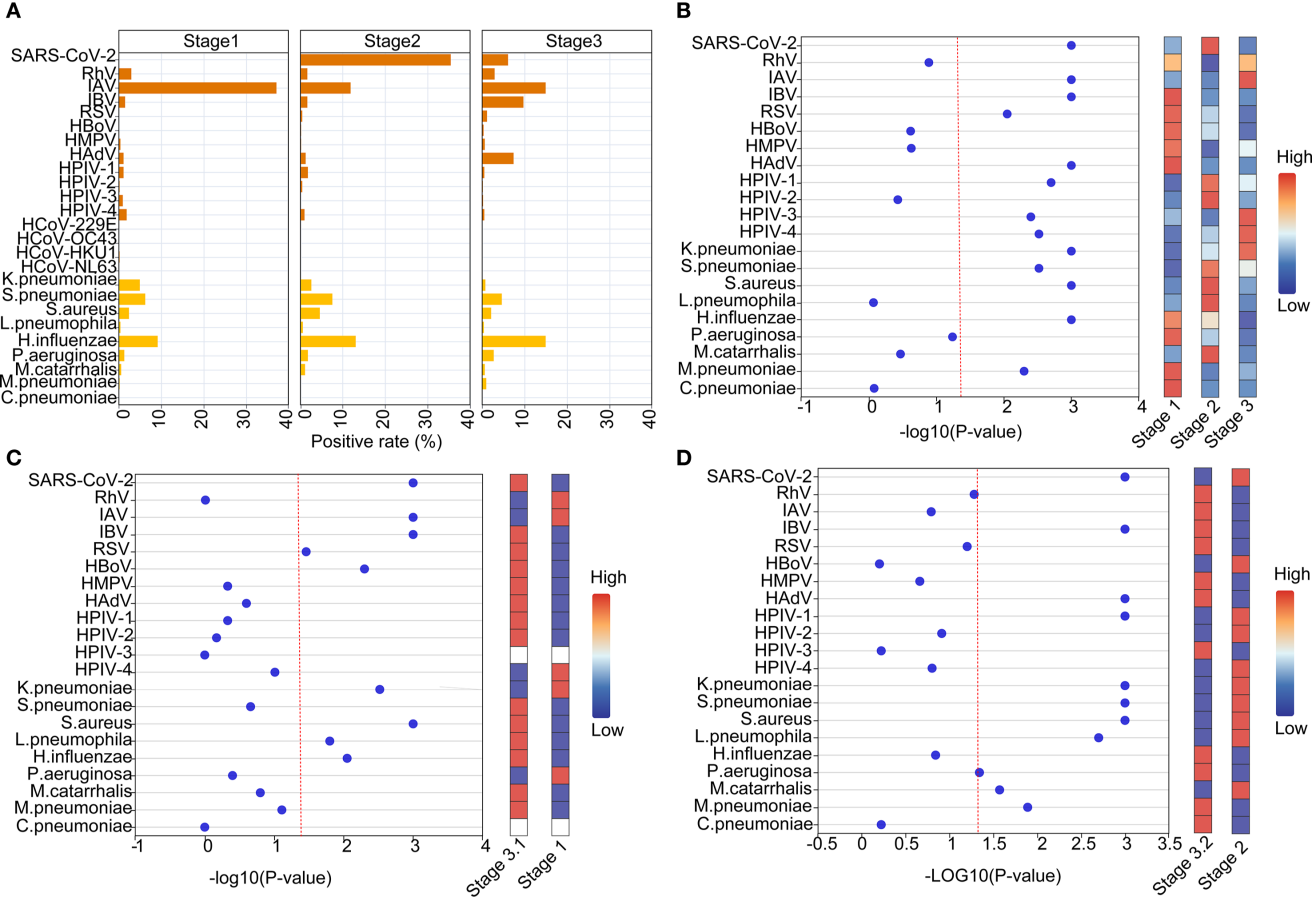
Comparative etiology and epidemiological patterns during and post NPIs of SARS-CoV-2. **(A)** The positive rates of different pathogens at different stages. **(B)** Normalized bubble heat-map shows the comparison of positive rates of different pathogens among the three stages. **(C, D)** Normalized bubble heat-map shows the comparison of positive rates of different pathogens between the indicated stages. The red dotted line indicated the p value of 0.05.

Overall, there were disparities in positivity rates of detected pathogens across the three stages, with significantly higher rates of Stage 2 than the other two Stages (**Supplementary Table S1**). In Stage 1, the most predominant respiratory pathogen was IAV (37.18%), followed by *H.influenzae* (9.16%), *S.pneumoniae* (6.23%), *K.pneumoniae* (4.95%) and RhV (2.93%) within the top 5 pathogens. Then SARS-CoV-2 became the dominate pathogen during Stage 2, accounting for an overall positivity rate of 35.44%. Except for SARS-CoV-2, *H.influenzae* (13.02%), IAV (11.81%), *S.pneumoniae* (4.61%) and *S.aureus* (4.56%) were within the top 5 pathogens. In Stage 3, IAV and *H.influenzae* possess the highest positivity rate *(*both 14.97%), followed by IBV (9.73%), HAdV (7.41%) and SARS-CoV-2 (6.09%), with an increase in the positivity rate for IBV and HAdV and compared to the Stage 1 and Stage 2 (**Figure 3A**, **Supplementary Table S1**). In detail, distinct epidemiological patterns were found for multiple pathogens including SARS-CoV-2, IAV, IBV, RSV, HAdV, HPIV-1, HPIV-3, HPIV-4, *K.pneumoniae*, *S.pneumoniae*, *S.aureus*, *H.influenzae* and *M.pneumoniae* among the three stages (**Figure 3B**, **Supplementary Table S1**). Considering the possible influence of season, we further divided Stage 3 into Stage 3.1 (Jul 2023–November 2023, corresponding to Stage 1) and Stage 3.2 (2023.12-2024.05, corresponding to Stage 2) for further analyses. The results showed that positivity rates of SARS-CoV-2, IAV, IBV, RSV, HBoV, *K.pneumoniae*, *S.aureus*, *L.pneumophila* and *H.influenzae* were statistically different between Stage 1 and Stage 3.1 (**Figure 3C**), and positivity rates of SARS-CoV-2, IBV, HAdV, HPIV-1, *S.pneumoniae*, *K.pneumoniae*, *S.aureus*, *L.pneumophila*, *P.aeruginosa* were statistically different between Stage 2 and Stage 3.2 (**Figure 3D**).

**Figure 4:**
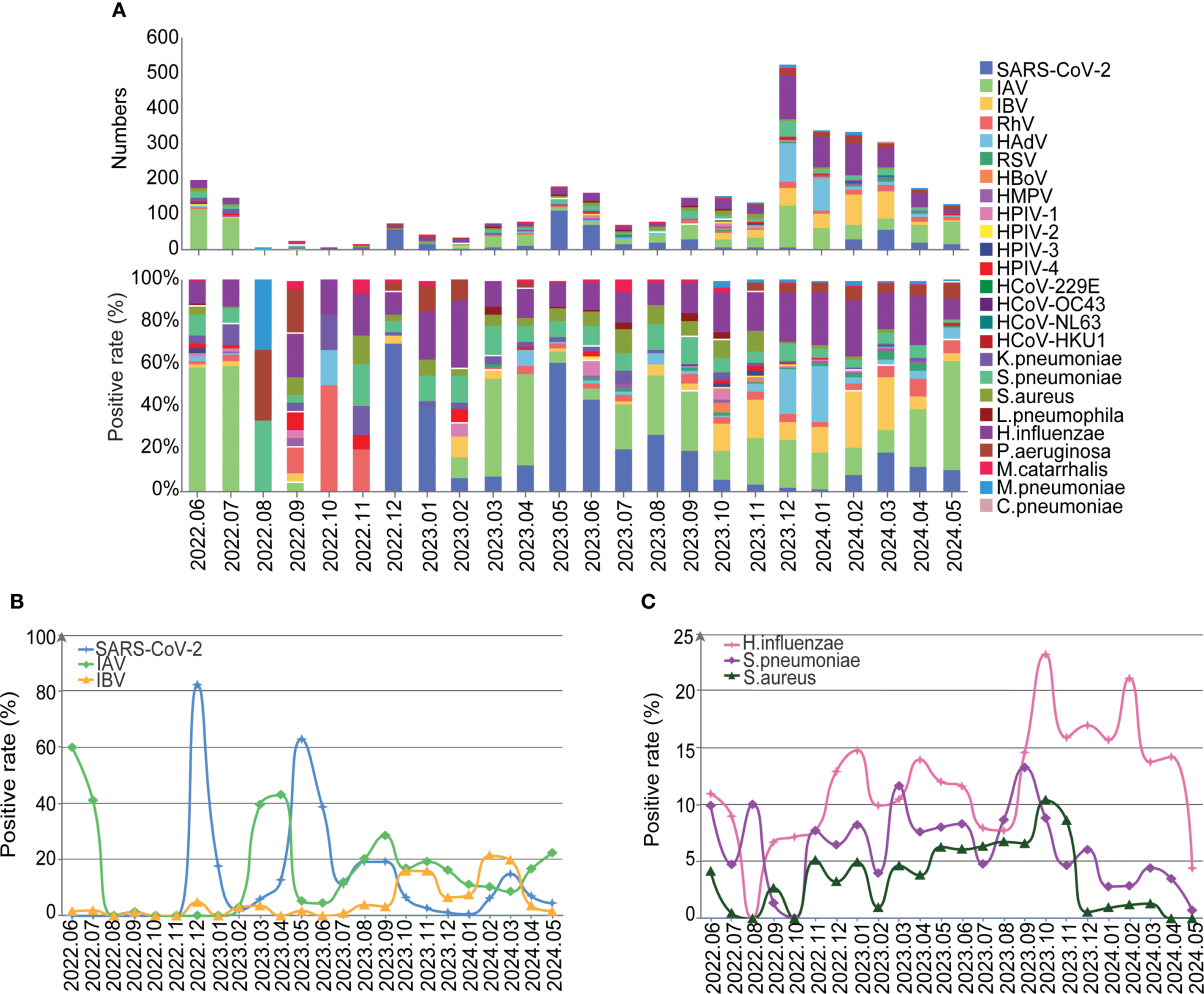
The detailed distribution and trends of the detected pathogens. **(A)** The etiology and compositions of pathogens by month. **(B)** The positive rate of SARS-Cov-2, IAV and IBV over time. **(C)** The positive rate of H.influenzae, S.pneumoniae and S.aureus over time.

Further analyses on the monthly data showed that positivity rates of SARS-CoV-2, IBV, HAdV and *H.influenzae* pathogens gradually increased with the termination of NPIs (**Figures 4A, B**). And We further conducted an interrupted time-series analysis on several important pathogens including SARS-CoV-2, IAV, IBV, HAdV, and *H. influenzae*. The analysis revealed that SARS-CoV-2 exhibited an immediate sharp peak following the lifting of NPIs, followed by a sustained downward trend, indicating that the infection wave triggered by the termination of NPIs subsided rapidly. Peaks for SARS-CoV-2 were found in December 2022 (82.26%), May 2023 (62.86%), September 2023 (19.21%) and March 2024 (14.77%), with decreasing positivity rates (**Figures 4A, B**, **Supplementary Figure 2**). IAV and IBV demonstrated distinct response patterns. IAV serves as the most predominant respiratory pathogen during the NPIs with peak prevalence in June (59.90%)-July (41.04%) 2022, and other peaks post NPIs were found in April 2023 (43.04%), August 2023 (28.48%) and May 2024 (22.30%) (**Figures 4A, B**). In addition, the positivity rates of pathogens such as influenza B, *H.influenzae*, *S.pneumoniae* and *S.aureus* also showed a fluctuating increasing trend (**Figure 4C**, **Supplementary Figure 2**). HAdV experienced an epidemic peak in December 2023 and January 2024, primarily affecting children, with 75.85% of pediatric cases attributed to HAdV during this period (**Figure 4**, **Supplementary Figures 2**, **3**). Overall, this analysis reveals the heterogeneous responses of different respiratory pathogens to changes in public health policies.

### Comparative etiology and epidemiological patterns between gender

**Figure 5:**
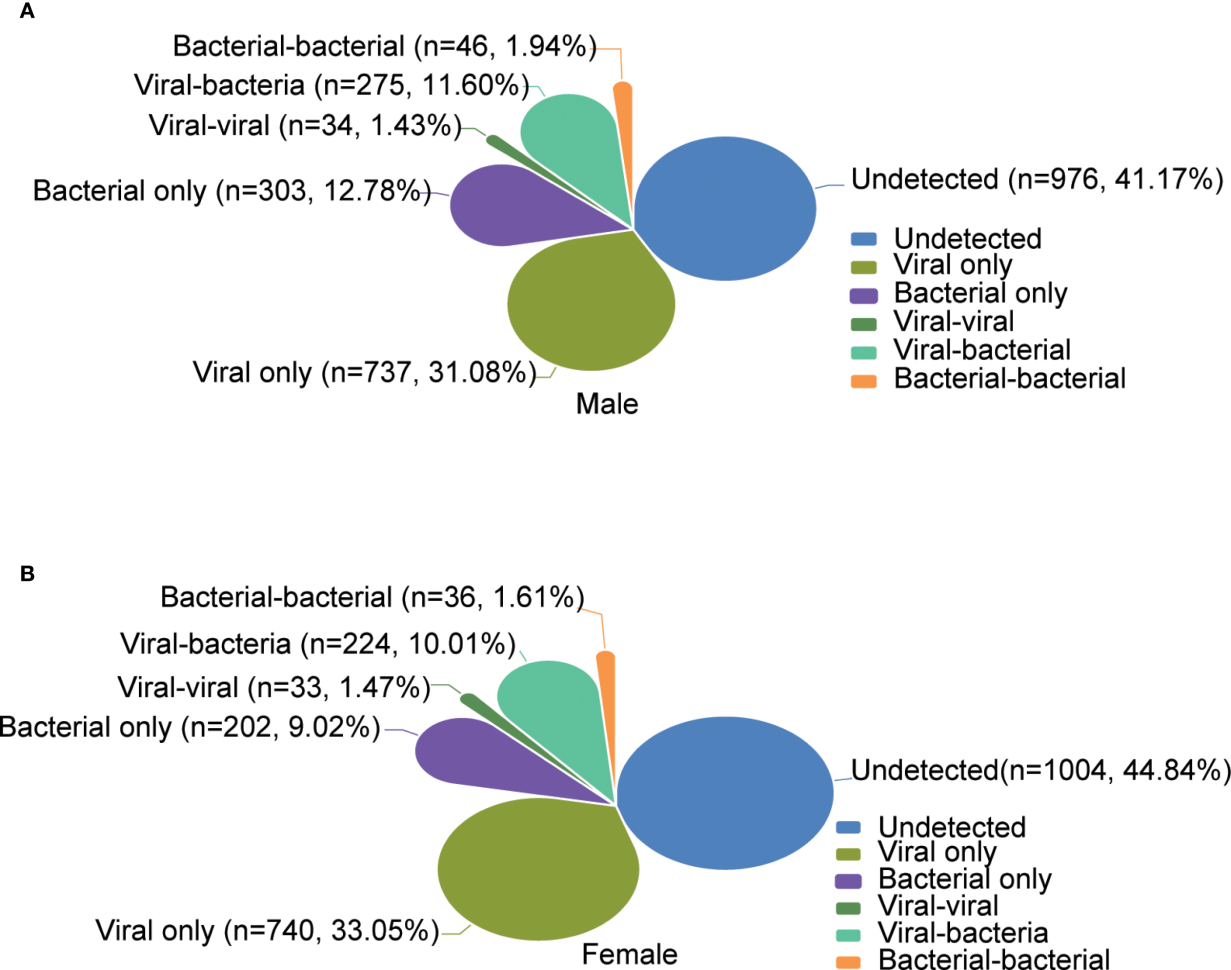
Comparative etiology and epidemiological patterns between gender. **(A, B)** show the overall distribution of the positivity rates of infection types for male and female, respectively.

The overall positivity rate of detected pathogens was significantly higher for the males (1395/2371, 58.84%) than the females (1235/2239, 55.16%). Among the males, the proportions of single viral infection, single bacterial infection, viral co-infection, bacterial co-infection, and co-infection between virus and bacteria were 31.08% (737/2371), 12.78% (303/2371), 1.43% (34/2371), 1.94% (46/2371), and 11.60% (275/2371), respectively. Among the females, the corresponding proportions were 33.05% (740/2239), 9.02% (202/2239), 1.47% (33/2239), 1.61% (33/2239), and 10.00% (224/2239), respectively (**Figure 5**). The top five pathogens detected in males and females were consistent with the overall distribution. Among viral pathogens, IAV (males: 16.87%, females: 17.33%), SARS-CoV-2 (males: 9.28%, females: 10.99%), IBV (males: 7.04%, females: 7.86%), HAdV (males: 6.83%, females: 4.42%), and RhV (males: 3.16%, females: 2.23%) were the most prevalent in males and females, and statistical difference was only found for HAdV (*P = 0.001*). As to bacterial pathogens, the top five in both males and females were *H.influenzae* (males: 16.20%, females: 11.61%), *S.pneumoniae* (males: 5.78%, females: 4.73%), *S. aureus* (males: 2.61%, females: 2.46%), *P.aeruginosa* (males: 2.61%, females: 2.14%), and *K.pneumoniae* (males: 1.73%, females: 1.34%), and statistical difference was only found for *H.influenzae* (*P < 0.001*) (**Supplementary Table S2**, **Supplementary Figure 3**). We conducted an analysis to explore the relationship between the Cycle Threshold (Ct) values of individual pathogens and gender. Our findings revealed no statistically significant differences in the Ct values of any pathogens when stratified by gender (**Supplementary Figure 5B**).

### Age-related etiology and epidemiological patterns

**Figure 6:**
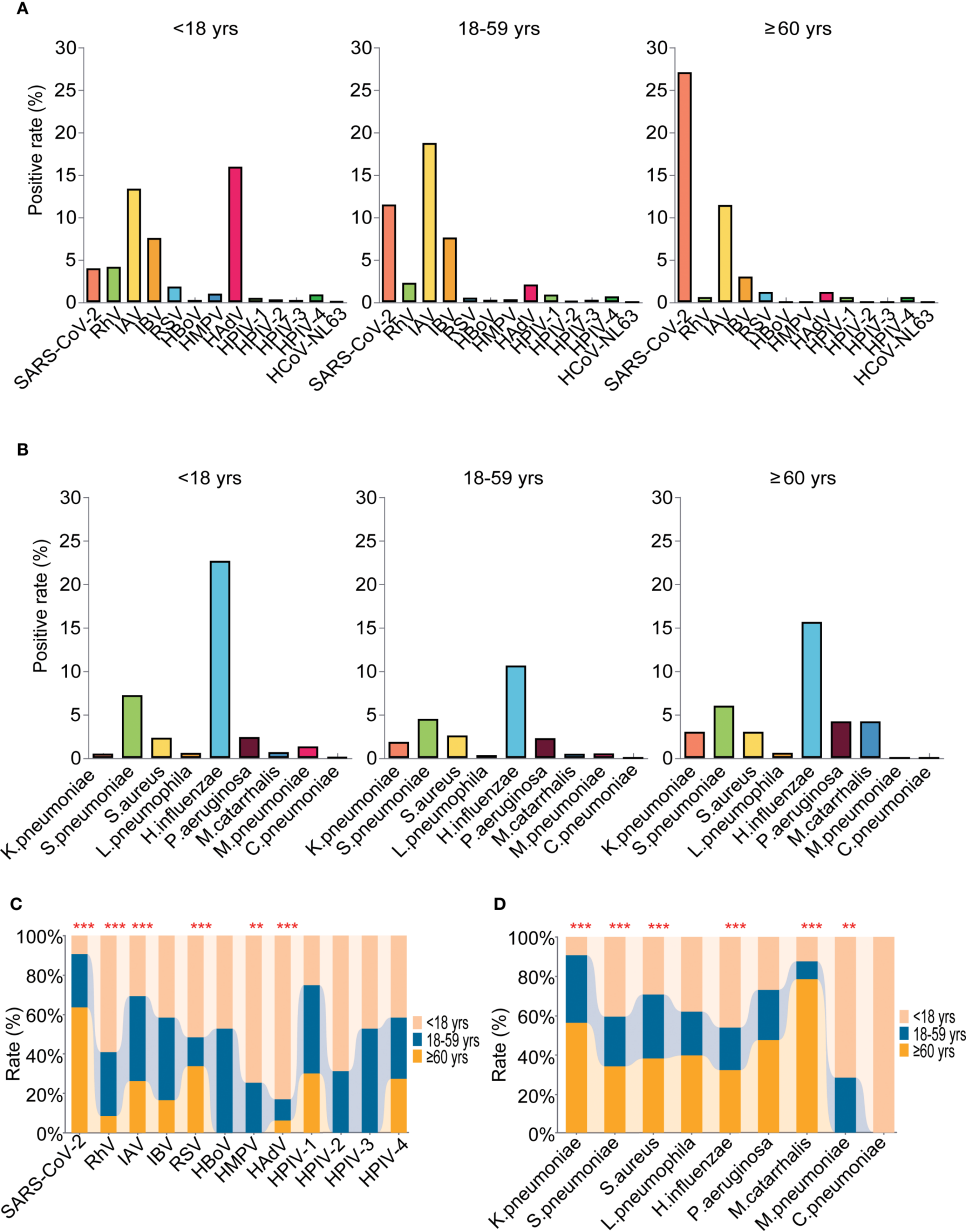
Age-related etiology and epidemiological patterns of the detected pathogens. **(A)** The positivity rate of different viruses in the three age groups. **(B)** The positivity rate of different bacteria in the three age groups. **(C)** Comparative analyses of positivity rates for each virus in the three age groups. **(D)** Comparative analyses of positivity rates for each bacteria in the three age groups. ** means P<0.01, *** means P< 0.001.

The highest positivity rate of detected pathogens was found in the elderly group 63.86% (95%CI: 56.29% - 71.43%), followed by the children’s group 62.76% (95%CI: 60.05% - 65.47%) and then the adult group at 54.58% (95%CI: 52.89% - 56.27%), and the positivity rates are higher in children and the elderly group than in adult group (*P =0.001*). As to the viral infection, the positivity rates were 47.22%(95%CI: 44.40% - 50.04%), 45.18% (95%CI:37.38% - 53.15%) and 43.20% (95%CI: 41.53% - 44.87%) for the children’s group, elderly group and adult group, respectively. However, for the bacteria infections, the positivity rates were in the order of children’s group (31.26%, 95%CI: 28.63% - 33.89%), elderly group (30.72%, 95% CI: 23.77% - 38.32%), and then the adult group (20.33%, 95% CI: 23.77% - 38.32%) (**Supplementary Table S3**). Further age-specific analyses showed that the top five most prevalent pathogens were different across age groups with the rank being *H.influenzae* (22.69%, 95%CI: 20.30% - 25.08%), HAdV (15.96%, 95%CI: 13.86% - 18.06%), IAV (13.38%, 95%CI: 11.40% - 15.36%), IBV (7.56%, 95%CI: 6.02% - 9.10%) and *S.pneumoniae* (7.23%, 95%CI: 13.86% - 18.06%) for the children group, and the prevalence of RhV, RSV, HMPV, HAdV, *S.pneumoniae, H.influenzae* and *M.pneumoniae* were significantly higher than the other two groups (**Figure 6**). For the adult group, the top five pathogens were IAV (18.76%, 95%CI: 17.47%- 20.05%), SARS-CoV-2 (11.51%, 95%CI: 10.42% - 12.60%), *H.influenzae* (10.64%, 95%CI: 9.56% - 11.72%), IBV (7.62%, 95%CI: 6.66% - 8.58%) and *S.pneumoniae* (4.50%, 95%CI: 1.38% - 2.32%), and the positivity rate of IAV was significantly higher than the other two groups (**Figure 6**). While in the elderly group, SARS-CoV-2 (27.11%, 95% CI: 20.40% - 34.70%), *H.influenzae* (15.66%, 95% CI: 10.24% - 22.21%), IAV (11.45%, 6.70% - 17.46%), *S.pneumoniae* (6.02%, 95% CI: 2.56% - 11.10%), and *P.aeruginosa* (4.22%, 95% CI: 1.37% - 8.58%) were within the top five, and the frequencies of SARS-CoV-2, *K.pneumoniae* and *M.catarrhalis* were found to be significantly higher (**Figure 6**, **Supplementary Table S3**). We further analyzed the association between Ct values of each pathogen and age. Our analyses revealed that the Ct values of IBV (P=0.036), HAdV (P=0.002), and RSV (P=0.01) were significantly lower in the pediatric group compared with the other two groups. Conversely, the Ct value of SARS-CoV-2 (P=0.049) was significantly higher in the elderly group than in the other two groups. No significant differences in Ct values were noted for the remaining pathogens(**Supplementary Figure 5C**).

### Seasonal etiology and epidemiological patterns

**Figure 7:**
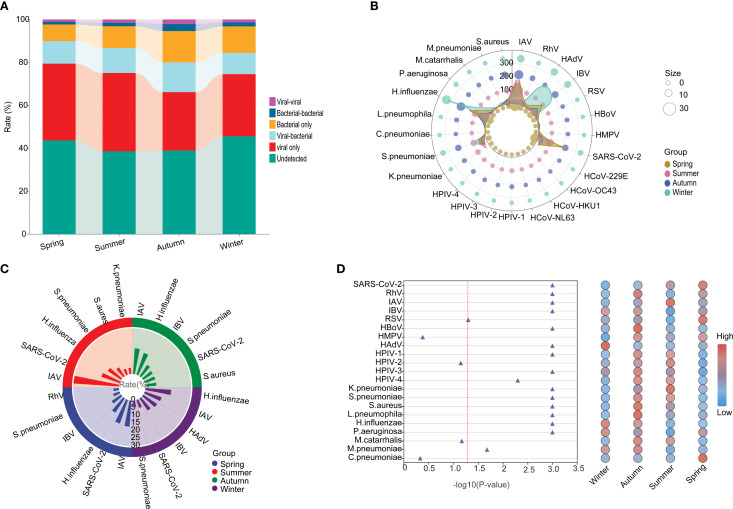
The seasonal etiology and epidemiological patterns. **(A)** General distribution of the infection types in each season. **(B)** Polar diagram representing the detailed distribution characteristics of 25 pathogens. **(C)** The seasonality distribution top 6 of viral positivity rate. **(D)** Normalized bubble heat-map shows the comparison of positivity rates of different pathogens across the four seasons. The red dotted line indicated the p-value of *0.05*.

The highest positivity rate of detected pathogens was found in summer (61.45%), followed by autumn (61.08%), spring (56.37%), and then winter (54.46%), For viral infection, the highest positivity rate was also found in summer (49.70%), followed by spring (47.37%), winter (43.24%), and autumn (40.23%). However, for the bacteria infections, the highest positivity rate was found in autumn (14.59%), winter (12.40%), summer (10.18%), and spring (8.95%) (**Figure 7A**). In detail, the top 5 prevalent respiratory pathogens were IAV (17.09%), SARS-CoV-2 (16.4%), *H.influenzae* (11.29%), IBV (7.4%) and *S.pneumoniae* (4.5%) in spring, IAV (29.82%), SARS-CoV-2 (12.61%), *H.influenzae* (10.55%), *S.pneumoniae* (7.03%) and *S.aureus* (4.12%) in summer, IAV (16.94%), *H.influenzae* (15.14%), IBV (9.01%), *S.pneumoniae* (7.57%) and SARS-CoV-2 (7.39%) in autumn, and *H.influenzae* (16.94%), IAV (11.67%), HAdV (10.94%), IBV (9.59%) and SARS-CoV-2 (4.38%) in winter (**Figures 7B, C**). The infection rates of SARS-CoV-2 were found to be significantly higher during the spring and summer months compared to the autumn and winter months (*P < 0.001*). Furthermore, a noteworthy surge in the prevalence of HAdV was observed in pediatric patients during the winter season. Overall, clear seasonal trends were evident for a range of respiratory pathogens with the exception of HMPV, HPIV-2, *M.catarrhalis* and *C.pneumoniae*, and many pathogens were distinctly more prevalent in summer and autumn than in spring and winter (**Figure 7D**).

### Co-infection patterns

**Figure 8:**
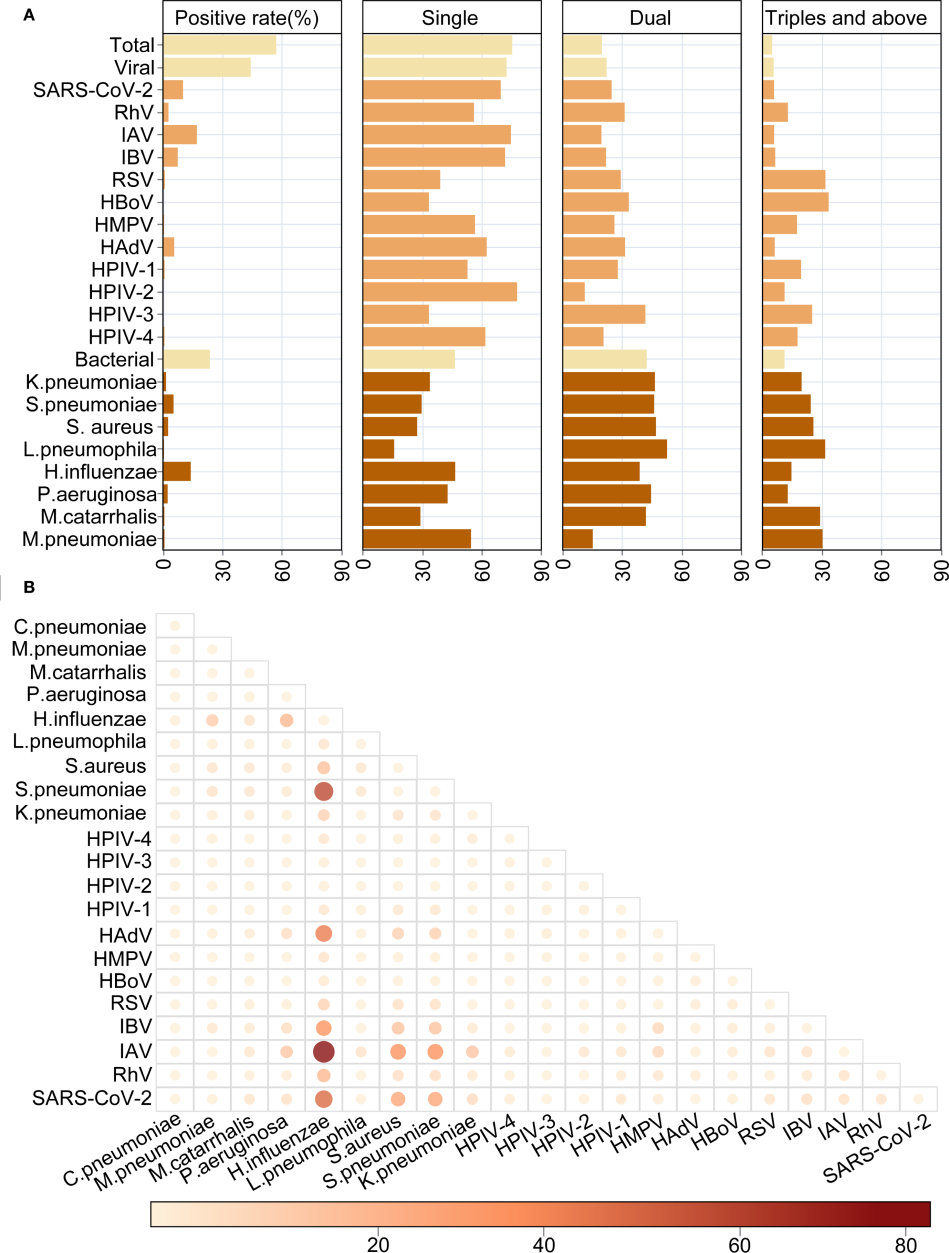
The characteristics of co-infections between respiratory pathogens. **(A)** The overall distribution of mono- or multi-infections. **(B)** Heat map shows the detailed co-infections between different respiratory pathogens.

Co-infection with multiple pathogens is a common phenomenon in acute respiratory infections. In our study, the overall co-infection rate was 24.62%, with 19.76% of cases involving two pathogens and 4.86% involving three or more (**Figure 8A**). Specifically, the viral co-infection rate was 23.44%, with the highest co-infection rates found for RhV (44.00%), HAdV (37.55%), SARS-CoV-2 (30.47%), IBV (28.28%), and IAV (25.38%) (**Figure 8A**). Among them, the most common viral co-infections were IAV and *H.influenzae*, followed by SARS-CoV-2 and *H.influenzae*, HAdV and *H.influenzae*, IBV and *H.influenzae*, SARS-CoV-2 and *S.pneumoniae*, IAV and *S.pneumoniae* (**Figure 8B**). Compared to viral co-infection, higher rates of bacterial co-infection were found (53.40 vs. 23.44%). Except for the viral-Among the top 10 infection pathogens, the highest co-infection rates were observed with bacterial pathogens, specifically *S.aureus* (72.65%), *S.pneumoniae* (70.37%), *K.pneumoniae* (66.20%), *P.aeruginosa* (57.27%) and *H.influenzae* (53.42%).

Then we conducted a thorough analyses of the co-infection patterns of HAdV, SARS-CoV-2, IAV, IBV, *H.influenzae, S.pneumoniae* and *S.aureus* across different periods, genders, ages, and seasons. The results showed that no significant differences were observed in the co-infection rates of several major pathogens at different stages. (**Supplementary Table S4**). Among them, the most frequent co-infections of IBV found to be SARS-CoV-2. However, the most commonly co-infection of SARS-CoV-2 was associated with *H.influenzae*. The co-infection rate of IAV in males is higher than that in females (29.50% vs 21.13%; *X^2^ = 6.843*, *P = 0.001*) (**Supplementary Table S5**), and the co-infections between IAV with *H.influenzae* were the most frequent. The co-infection rates of multiple pathogens differ across various age groups. In the pediatric group, the rates of co-infections involving SARS-CoV-2, IAV, and *H.influenzae* were 54.17%, 37.27% and 60.44%, respectively, and all of them were significantly higher than the other two groups (**Supplementary Table S6**). Moreover, the co-infections between SARS-CoV-2 and *H.influenzae*, IAV and *H.influenzae*, HAdV and *H.influenzae* in the pediatric group were the most frequent. Additionally, in terms of seasonality, both IAV (37.23%) and IBV (48.00%) exhibited the a significantly higher co-infection rates in autumn (**Supplementary Table S7**), both most frequently co-infected with *H.influenzae*.

## Discussion

In this two years’ prospective cohort study, we enrolled 4610 patients from the fever clinic of Shenzhen Third People’s Hospital and analyzed the changing causal and epidemiological characteristics of ARTIs during and post NPIs against SARS-CoV-2. We further described the patterns of infection and co-infection of 16 viral and 9 bacterial pathogens associated with age, gender and seasons. In this study, the pathogen epidemics of the two years were divided into three stages based on the SARS-CoV-2 epidemics to investigate the longitudinal etiology and epidemiological patterns of ARTIs during and post NPIs of SARS-CoV-2 in Shenzhen, China. Stage 2 was found to have the highest positivity rate of pathogen, and SARS-CoV-2 also had the highest positivity rate of in this stage. This may be due to the fact that the level of immunity to various pathogens was lower in the population during the period of NPIs (Rana et al., 2021), and therefore Stage 2 presents an outbreak epidemic with SARS-CoV-2 as the main pathogen. In addition, the positivity rates of multiple pathogens showed significant differences between three stages (Xiao et al., 2021). Therefore, the NPIs has changed the epidemiological spectrum of respiratory pathogens to some extent (Rana et al., 2021; Li et al., 2022; Wei et al., 2024).

During various NPIs stage for SARS-CoV-2, there have been substantial fluctuations in the circulation of ARTIs: In the strict NPI stage (Stage 1), IAV exhibited a dominant prevalence, while other viral activities were suppressed, and bacterial infection rates remained stable. In the post-NPI relaxation stage with SARS-CoV-2 outbreaks (Stage 2), SARS-CoV-2 became the predominant pathogen, with a positivity rate of 35.44%. In the Stage 3, which is characterized by a regular epidemic, IAV and *H. influenzae* began to rebound, accompanied by a significant increase in the positivity rates of other viruses and bacteria, including HAdV and IBV. The majority of viruses exhibited elevated detection rates following the implementation of NPIs. Conversely, bacterial infection rates remained stable, while co-infections with viruses demonstrated a notable increase (resulting in an overall co-infection rate of 24.62%).

Ecologically, viral interference and super-infection likely contributed to these shifts. Viral interference may have occurred when dominant pathogens (e.g., SARS-CoV-2 in Stage 2) suppressed the circulation of other viruses, with reduced interference post-NPIs enabling the resurgence of IAV, HAdV, and IBV in Stage 3. These findings decisively address key knowledge gaps regarding the circulating characteristics of common respiratory viruses in the post-pandemic era and provide clear insights into virus-specific factors of circulating patterns. These factors might include environmental stability, infectivity, transmissibility, population immunity, and reservoirs, as well as possible virus-virus interaction (Kuitunen et al., 2022).

We revealed that ARTIs elicited similar symptoms in both children and adults, including fever, cough, rhinorrhea, sore throat, and myalgia. The prevalence of fever was higher in children and the symptoms reported were more varied for adults. This discrepancy could be attributed to younger children’s potential inability to accurately convey their symptoms. We showed an overall positivity rate 57.05%, which was comparable to previous studies (Arancibia et al., 2014; Zhang et al., 2023). Similar with a nationwide multi-center study in China (2009 to 2019) (Li et al., 2021), viral infection (44.32%) was also found to be more frequent than bacterial infection (23.60%) in our study, while both rates were higher in our study. For the remaining samples with undetermined pathogens (approximately 40%), various factors could be involved, including sample collection, load of pathogens, and coverage of the detected pathogens. These cases should not be overlooked, and more sensitive detection techniques or sequencing methods could be employed to investigate potential pathogen “X”, especially when a sudden increase in unknown causes is observed.

Several studies have reported a significant decrease in the positivity rate of IFV during the SARS-CoV-2 pandemic, such as a sharp decrease from 59.5% to 12.3% (Cullinan et al., 2022), and only a positivity rate of 4.3% in Ireland during the period from 2021 to 2022 (Brady et al., 2023). The largest decrease of annual cumulative positivity rate for IFV was observed in China with a reduction of 87.6% (from 9.94% to 1.23%) (Li et al., 2022). Consistent with other studies, the IFVs (including IAV and IBV) dominated the respiratory infections (24.53%) (Ren et al., 2009; Lu et al., 2013). Our results were demonstrated that after the end of the epidemic, IFV resumed its original epidemiological pattern. In addition, the samples did not include SARS-CoV-2 positive cases until December 2022 due to the implementation of the SARS-CoV-2 epidemic prevention and control measures. Our results found that, positivity rates of most viral pathogens have increased after the termination of NPIs, it may be due to prolonged absence of exposure to and infection with the respiratory pathogens, lack of stimulation of the body’s immune system by the pathogen, and reduced levels of herd immunity caused disease epidemics when re-exposed to the pathogen (Huang et al., 2022). The three infection epidemics of SARS-CoV-2 in one year, outbreaks in December 2022, May 2023, and August-September 2023, are the same as the trend of SARS-CoV-2 infection epidemics released by China CDC and WHO (COVID-19 cases | WHO COVID-19 dashboard).

Sex has been shown to be a factor influencing respiratory infections and their severity (Kadel and Kovats, 2018). Strong epidemiological evidence now exists that gender is an important biologic variable in immunity (Wilkinson et al., 2022). Several studies have shown that respiratory infections are associated with the influence of physiological gender, with differences in innate immune cells and differences in sex hormone secretion between men and women during the life cycle, with different susceptibility to disease (Arancibia et al., 2014; Zhang et al., 2023). Sex differences in innate immune cells may influence both the pro-inflammatory/effector phase and the resolution/tissue repair phase, which are crucial for the host response to respiratory pathogens infections (Kadel and Kovats, 2018; Silveyra et al., 2021). In addition, sex differences in lung development, structure, and function have been identified (Sathish et al., 2015; Lindgren et al., 2022). The lungs of human females are smaller than those of males of the same heil cells express sex steroid receptors, and their functional responses to different hormonal environments may influence the immune response and modulate infection severity (Thurlbeck, 1982; Sathish et al., 2015). Consistent with these studies, the overall positivity rate was also found to be higher in males than females (58.84% vs 55.16%, *P < 0.001*) in our study. Specifically, the prevalence of HAdV infection was more frequent in males than in females (6.83% vs 4.42%) within the viral pathogens, and also the *H.influenzae* within the bacterial pathogens (16.20% vs 11.61%). Some studies have indicated that the incidence of IAV, RSV, and SARS-CoV-2 is higher in males than in females, potentially due to the influence of estrogen receptor signaling on respiratory infections (Glezen et al., 1971; Karlberg et al., 2004). Our observations also revealed that the infection rates of HAdV and *H.influenzae* were higher in males than females, which may similarly be influenced by estrogen receptor signaling (Karlberg et al., 2004). Nevertheless, the specific mechanisms underlying these differences need further investigation.

ARTIs represent the most prevalent cause of disease in individuals across all age groups, characterized by high morbidity and mortality rates with a significant global health concern (Global burden of 369 diseases and injuries in 204 countries and territories, 1990-2019: a systematic analysis for the Global Burden of Disease Study 2019 - PubMed), and a number of studies have demonstrated that the etiology of ARTIs varies with age (Ruuskanen et al., 2011; Zar et al., 2016; Pneumonia Etiology Research for Child Health (PERCH) Study Group, 2019; Li et al., 2021; Snoeck et al., 2021). Our results showed that the pathogen positivity rate was higher in elderly group (63.86%) and children’s group (62.76%) than adult group (54.58%) with statistical significance (*P=0.001*). These age-dependent patterns may also be associated with increased susceptibility to infection in childhood and elderly people, mainly due to a lower specific immune response, both quantitatively and functionally (Prendergast et al., 2012). Infections in children are more complex, suggesting the need for early clinical consideration of the possibility of bacterial-viral co-infection and avoidance of bacterial infections missed by single antiviral therapy (Prendergast et al., 2012). The high prevalence of SARS-CoV-2 and *P. aeruginosa* infections, in conjunction with the high prevalence of underlying disease, necessitates the prioritization of surveillance for co-infection with these two pathogens. Furthermore, heightened vigilance is imperative to address the risk of severe disease, and prompt intervention is essential. Specifically, the declining physical function and basic illnesses may increase the susceptibility of the elderly (Prendergast et al., 2012; Liu et al., 2023), and lower levels, affinity and less diversity of T cell-dependent antibody responses in children than adults may also account for these age-dependent patterns (Prigge et al., 2020). IAV and SARS-CoV-2 were established as the foremost viral pathogens in adults and the elderly, concordant with contemporary literature (Li et al., 2021; Seon et al., 2023). Interestingly, previous studies demonstrated that RSV was found to be the most prevalent respiratory pathogen in children with ARTIs (Li et al., 2021; Wang et al., 2021). In our study, *H.influenzae* was identified as the dominant pathogen, followed by HAdV and IAV in childhood. Furthermore, we observed that the rates of co-infections involving SARS-CoV-2, IAV, and *H.influenzae* were 54.17%, 37.27%, and 60.44% in the pediatric group, respectively, which is higher than in the other two age groups. This difference may be attributed to the underdeveloped immune system of children and the types of environments they experienced, such as kindergartens and schools, where cross-infections among pathogens are more likely to occur.

Previous studies have found that the seasonality of various viruses differs across different countries, research periods, and regions. Understanding the relationship between the seasonality of different viruses and meteorological factors is key to successfully implementing prevention and control programs (Li et al., 2019). Studies have shown that temperature and humidity have an effect on the prevalence of respiratory pathogen infections, so the seasonality of respiratory pathogens has been widely described (Audi et al., 2020; Moriyama et al., 2020; Hamid et al., 2023), and previous studies have also shown that the virus-positive rate was higher in southern China than in northern China, with relatively small monthly differences (Li et al., 2019; Li et al., 2021). Outbreak dynamics driven by climatic factors (mainly temperature) partially explained the overall detection rates and seasonal patterns of respiratory viruses, and these relationships varied with latitude (Yu et al., 2013; Baker et al., 2019; Li et al., 2021; Snoeck et al., 2021). In our study, the overall positivity rates of detected pathogens were significantly higher in summer and autumn than those in winter and spring, with the most frequent of viral infections in the summer and the bacterial infections in the autumn. This result differs from observations from the northern China with the higher prevalence in winter and spring (Li et al., 2021). Additionally, the co-infection of IAV and IBV with other pathogens tends to be more severe in the summer and autumn. This may be attributed to the rainy and windy conditions in the Shenzhen area during these seasons, which create a humid environment. Moreover, strong winds can lower surface body temperature, leading to vasoconstriction of the respiratory mucosa and suppression of the immune response, thereby increasing susceptibility to infections (Xu et al., 2021). Notably, SARS-CoV-2 was shown to be the most common cause of respiratory illness in spring, and a notable increase in HAdV positivity rates was observed in winter. Previous research has shown that humidity is positively correlated with HAdV, it is relatively stable at high humidity levels, and the higher humidity in the Shenzhen area during spring may be a significant reason for the elevated incidence of HAdV infections (Pica and Bouvier, 2012; Xu et al., 2021). It is imperative to formulate seasonal prevention and control strategies in conjunction with seasonal patterns. For instance, in the Shenzhen area, ventilation and disinfection measures should be augmented during the summer months to mitigate viral transmission. Concurrently, clinical vigilance for bacterial infections should be heightened in the autumn. These strategies are crucial to address the heightened prevalence of viral infections during summer months and the emergence of active bacterial infections in the autumn.

The reported prevalence of co-infection between pathogens varies significantly among different studies, ranging from 5.0% to 62.0% (average of 23.0%), and is associated with heightened risks of hospitalization and severe disease (Goka et al., 2014). Viral and bacterial co-infections are most prevalent, driven by factors such as respiratory microbiota imbalances, cell apoptosis, and inflammatory mediator dysregulation, which can suppress respiratory epithelial immune function (Liu et al., 2023). In our cohort, 24.62% of patients presented with co-infections, with the most common co-infections involved IAV and *H.influenzae*, followed by SARS-CoV-2 and *H.influenzae*, HAdV and *H.influenzae*, and IBV and H.influenzae. Several studies have also identified IFV co-infecting with *H.influenzae* as the most frequent scenario (Goka et al., 2013; Li et al., 2015). IFV infection coupled with other pathogens has been found to be associated with risks of increased severity and mortality (Goka et al., 2013; Kash and Taubenberger, 2015). As shown by one study, viral neuraminidase of IFV can augment bacterial adherence by inducing TGF-β, thereby elevating the risk of bacterial superinfection (Kash and Taubenberger, 2015). According to a recent study, bacterial co-infections were observed in 29.8% of hospitalized COVID-19 patients, and the presence of comorbidities further elevated the risk of severe disease and mortality (Zhang et al., 2020). The presence of co-infection can serve as a prognostic indicator, prompting enhanced monitoring and intervention strategies for affected patients, such as early hospitalization and intensive care. Our data has also revealed a notable coexistence of *H.influenzae* and SARS-CoV-2, which is of concern during the treatment of SARS-CoV-2 infections. The study’s findings underscore the necessity of employing a multifaceted testing approach in clinical settings.

This study has several limitations that should be noted. Firstly, the study sample was limited to patients attending the fever clinic, the hospitalized individuals were not included. Secondly, the sample collection period during strict NPIs was relatively short. Thirdly, the study is a single-center study, which may limit the representativeness.

In conclusion, we conducted an in-depth longitudinal investigation into the etiology and epidemiological patterns of ARTIs during and post NPIs of SARS-CoV-2 in Shenzhen, China, based on a two years’ prospective cohort study of all ages. Our results showed that the etiology and epidemiological patterns of ARTIs during and post NPIs of SARS-CoV-2 in Shenzhen have changed overtime, and sex, age and seasonal patterns were also found. These findings are vital for the early and rapid detection of various respiratory pathogens, understanding of susceptible populations, and rational vaccination. Furthermore, the emergence of alternating epidemics of SARS-CoV-2 and influenza viruses necessitates meticulous surveillance. The implementation of vaccination programs, such as the influenza vaccination initiative, and the dissemination of pandemic alerts to susceptible populations well in advance are of paramount importance. The findings could provide useful information for the public health measures and the clinical management of respiratory infections.

## Data Availability

The raw data supporting the conclusions of this article will be made available by the authors, without undue reservation.

## Supplementary material

Supplementary Figure 1 Baseline characteristics of the cohort. **(A)** Characteristics and distribution of the sample at different stages. **(B)** Clinical presentation features of different infection types. **(C)** Clinical presentation features of children (age < 18 years). **(D)** Clinical presentation features of adults and the elderly (age ≥ 18). Total represents all patients in the group, Viral represents patients infected with viruses. Bacterial represents patients infected with bacteria, chlamydia and mycoplasma. Viral-bacterial represents patients with viral and bacterial co-infections. Undetected represents patients who tested negative for the 25 pathogens.

Supplementary Figure 2 Impact of pandemic control policy modifications on respiratory pathogen prevalence with interrupted time-series analysis.

Supplementary Figure 3 The monthly distribution and trends of each pathogen. **(A–C)** show the positivity rate of indicated pathogens over time.

Supplementary Figure 4 Comparison of different pathogens between male and female. **(A)** Comparative analyses of positivity rates for each viral pathogens. **(B)** Comparative analysis of positivity rates for each bacterial pathogens.

Supplementary Figure 5 Pathogen Ct value analysis **(A)** Distribution of Ct values by pathogen **(B)** Distribution of Ct values of each pathogen across different genders **(C)** Distribution of Ct values of each pathogen among different age groups.
