# Supplementary figures and images for: The dynamic etiology and epidemiological patterns of acute respiratory tract infections during and post non-pharmacological interventions of SARS-CoV-2 in Shenzhen, China: a two years’ prospective cohort study from June 2022
Source: Front Cell Infect Microbiol. 2025 Sep 19;15:1599536. doi: 10.3389/fcimb.2025.1599536 (PMC12491313; doi:10.3389/fcimb.2025.1599536)

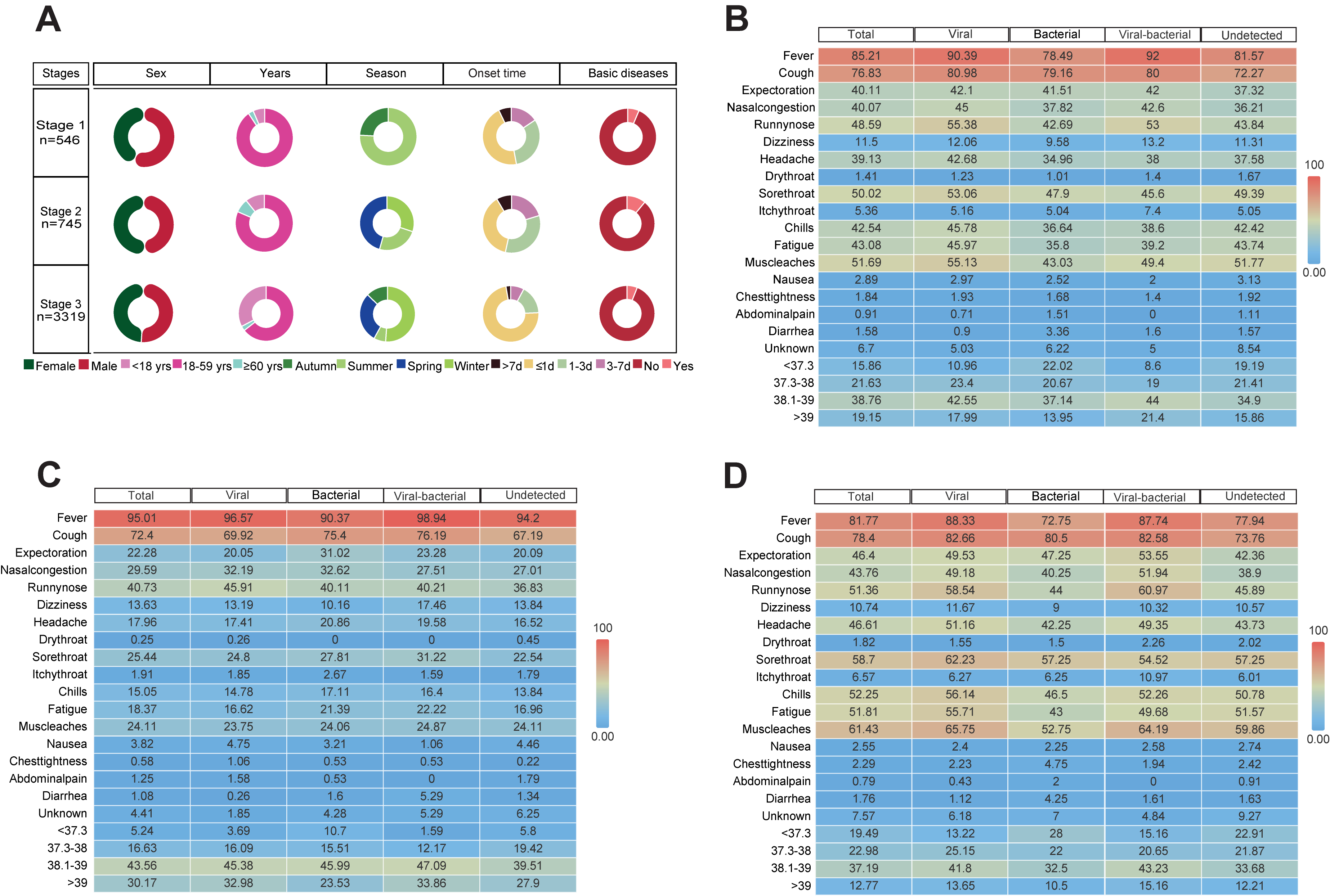

Supplement: Supplementary Figure 1 — Baseline characteristics of the cohort. (A) Characteristics and distribution of the sample at different stages. (B) Clinical presentation features of different infection types. (C) Clinical presentation features of children (age < 18 years). (D) Clinical presentation features of adults and the elderly (age ≥ 18). Total represents all patients in the group, Viral represents patients infected with viruses. Bacterial represents patients infected with bacteria, chlamydia and mycoplasma. Viral-bacterial represents patients with viral and bacterial co-infections. Undetected represents patients who tested negative for the 25 pathogens. [file Image1.tif]

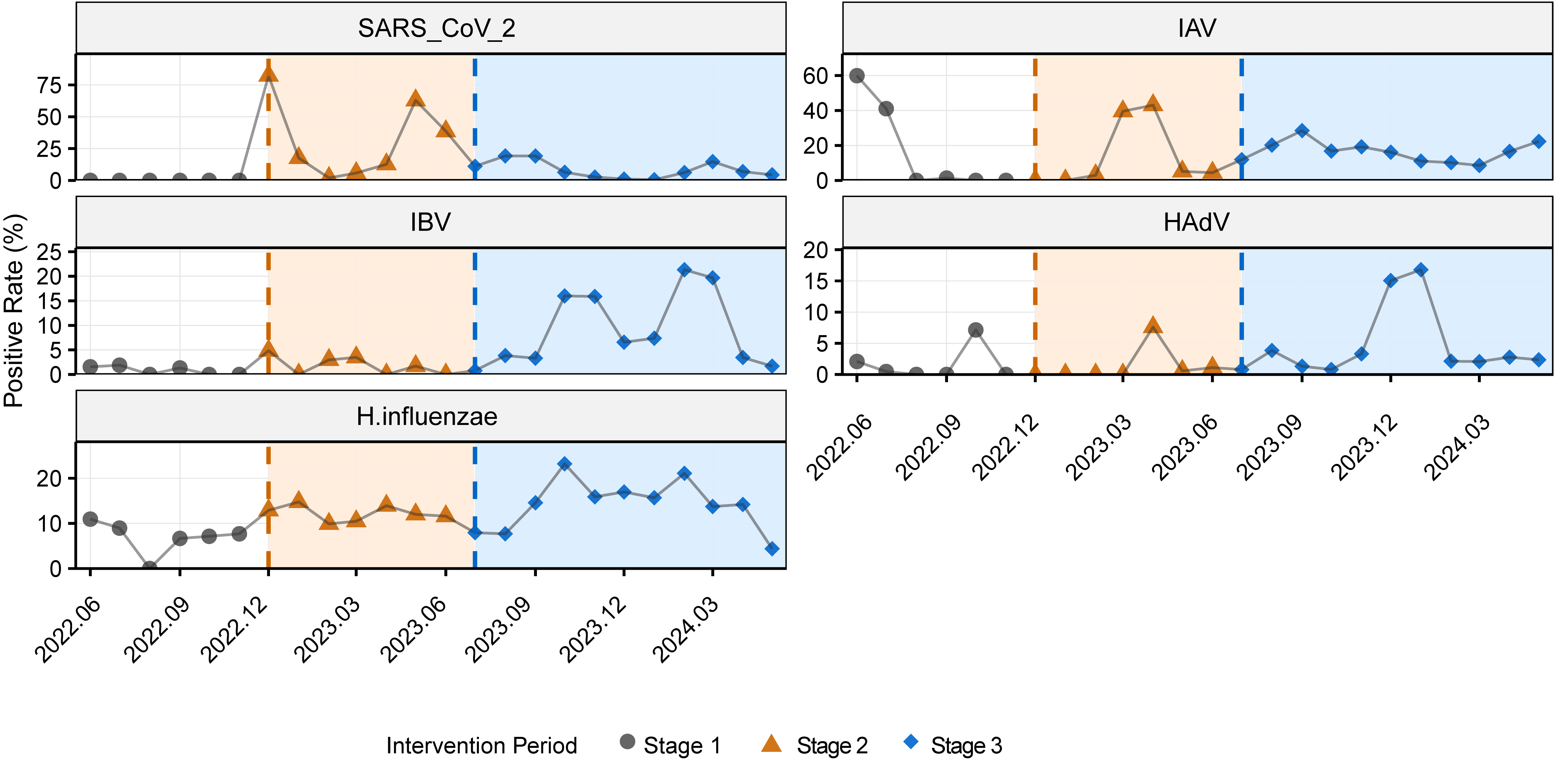

Supplement: Supplementary Figure 2 — Impact of pandemic control policy modifications on respiratory pathogen prevalence with interrupted time-series analysis. [file Image2.tif]

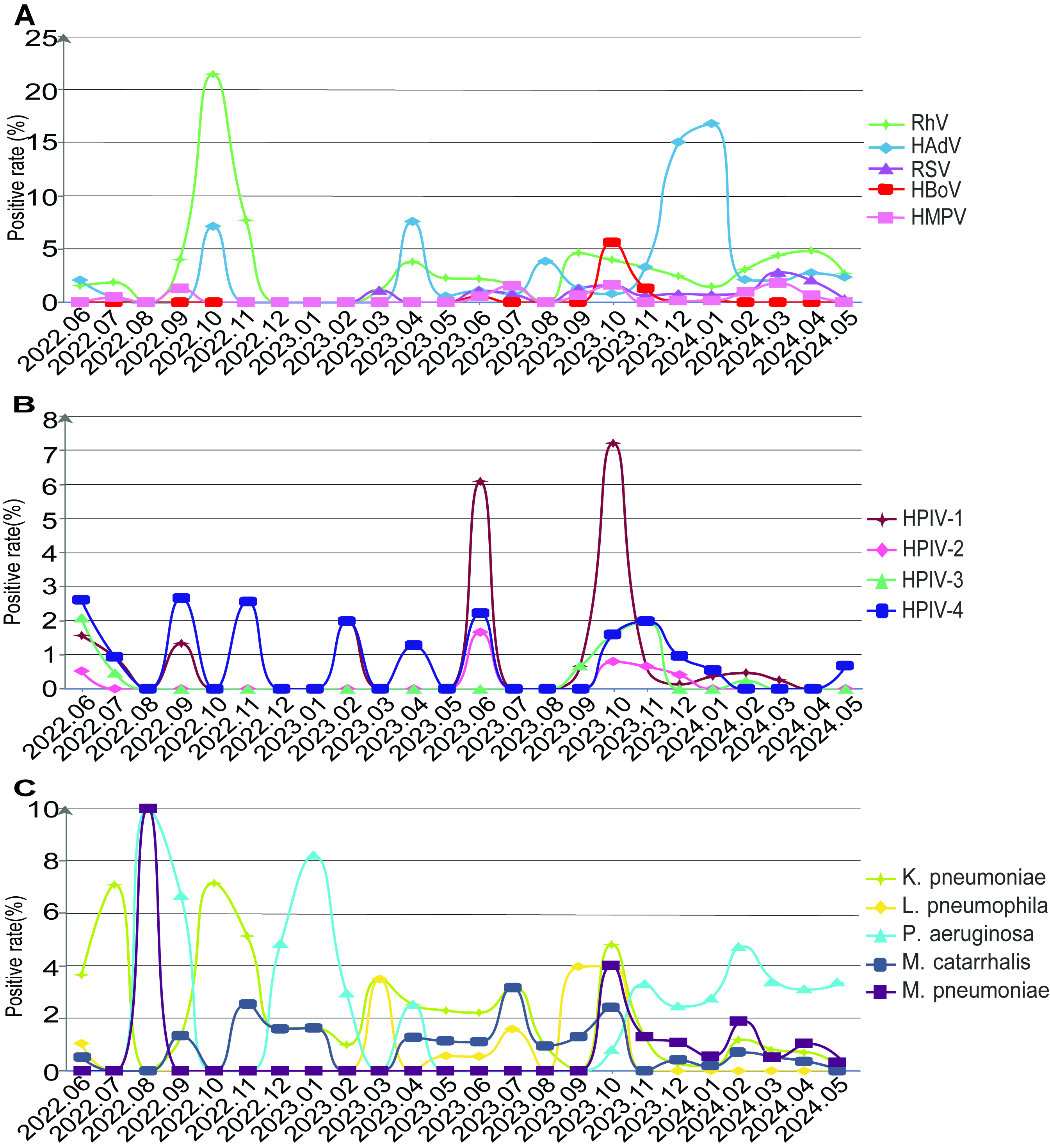

Supplement: Supplementary Figure 3 — The monthly distribution and trends of each pathogen. (A–C) show the positivity rate of indicated pathogens over time. [file Image3.tif]

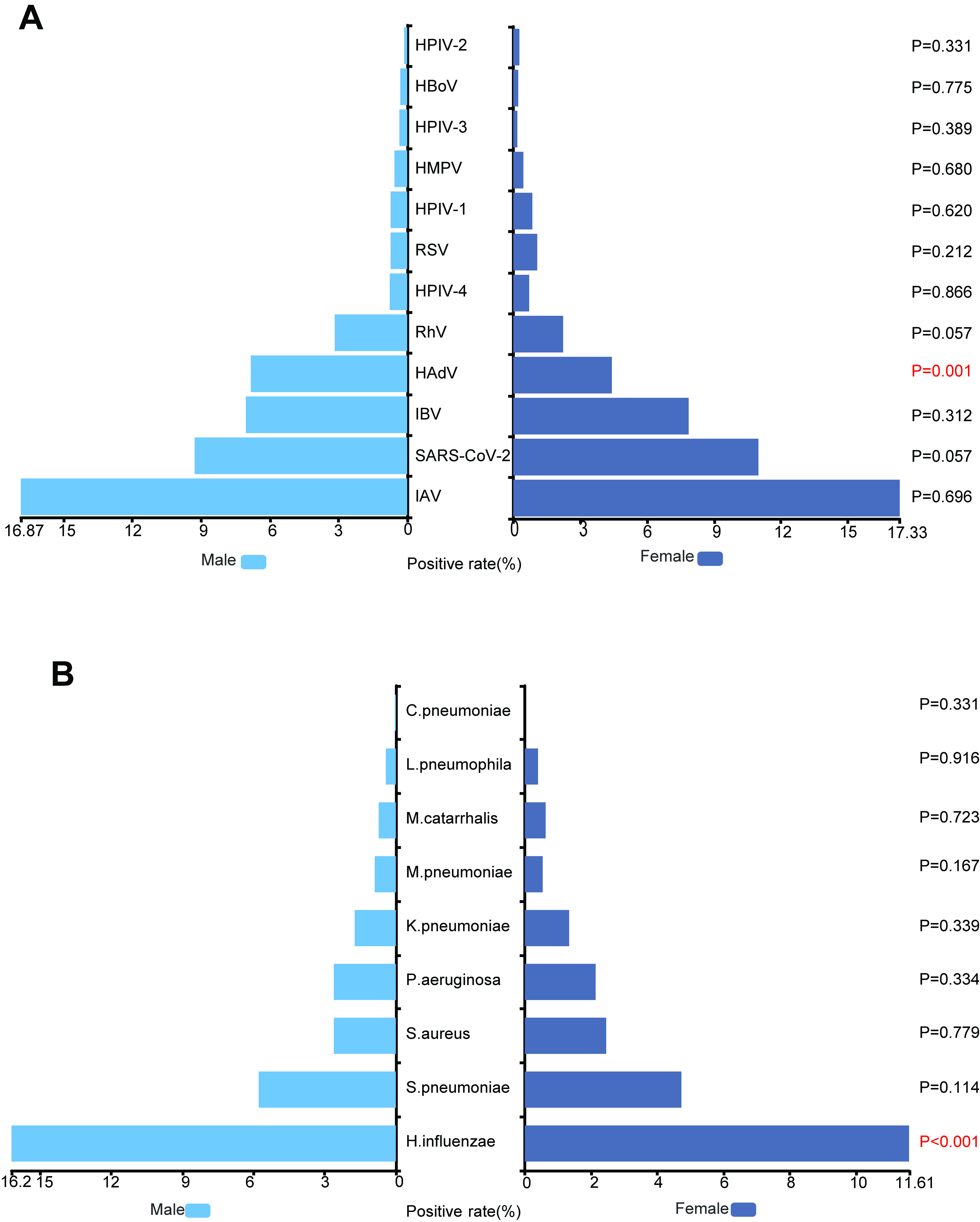

Supplement: Supplementary Figure 4 — Comparison of different pathogens between male and female. (A) Comparative analyses of positivity rates for each viral pathogens. (B) Comparative analysis of positivity rates for each bacterial pathogens. [file Image4.tif]

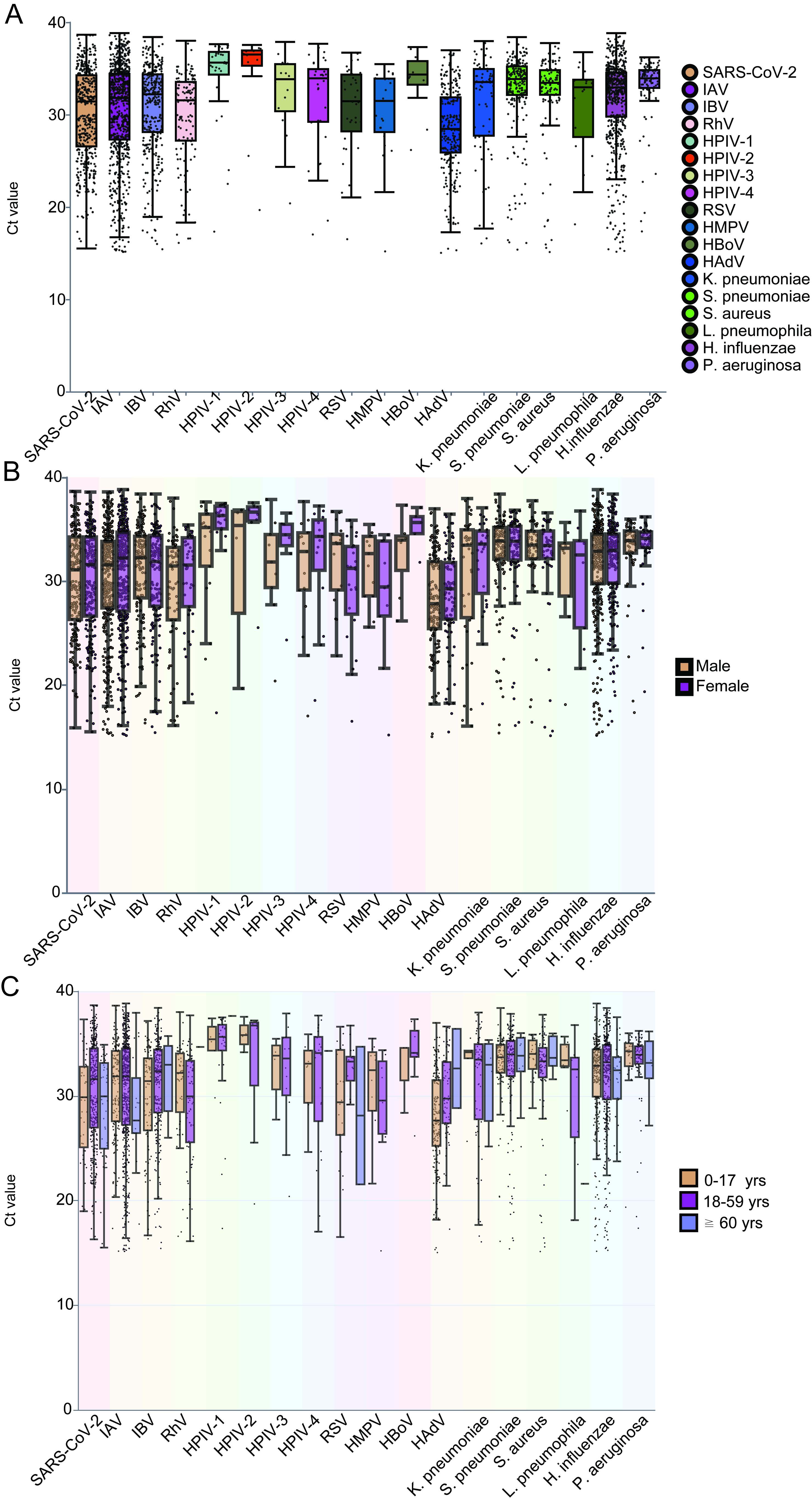

Supplement: Supplementary Figure 5 — Pathogen Ct value analysis (A) Distribution of Ct values by pathogen (B) Distribution of Ct values of each pathogen across different genders (C) Distribution of Ct values of each pathogen among different age groups. [file Image5.tif]
