# Supplementary material for: The dynamic etiology and epidemiological patterns of acute respiratory tract infections during and post non-pharmacological interventions of SARS-CoV-2 in Shenzhen, China: a two years’ prospective cohort study from June 2022
Source: Front Cell Infect Microbiol. 2025 Sep 19;15:1599536. doi: 10.3389/fcimb.2025.1599536 (PMC12491313; doi:10.3389/fcimb.2025.1599536)
Supplement: Supplementary file 6 [file Table1.docx]

Table S1. The positive distribution of respiratory pathogens in different stages.

| **Pathogens** | **Stage1**  **(%)** | **Stage2**  **(%)** | **Stage3**  **(%)** | **X^2^** | **Raw P-value** | **​FDR adjusted P​** |
| --- | --- | --- | --- | --- | --- | --- |
| Overall positive rate | 56.23 | 65.23 | 55.35 | 24.443 | ​0.001​ | ​0.001​ |
| Viral positive rate | 44.87 | 52.62 | 42.36 | 26.008 | ​0.001​ | ​0.001​ |
| SARS-CoV-2 | 0 | 35.44 | 6.09 | 646.446 | ​0.001​ | ​0.001​ |
| RhV | 2.93 | 1.61 | 2.92 | 4.082 | 0.13 | 0.195 |
| IAV | 37.18 | 11.81 | 14.97 | 180.621 | ​0.001​ | ​0.001​ |
| IBV | 1.47 | 1.61 | 9.73 | 90.376 | ​0.001​ | ​0.001​ |
| RSV | 0 | 0.4 | 1.14 | 9.361 | ​0.009​ | ​0.020​ |
| HBoV | 0 | 0.13 | 0.33 | 2.528 | 0.238 | 0.273 |
| HMPV | 0.37 | 0.13 | 0.6 | 2.908 | 0.234 | 0.273 |
| HAdV | 1.1 | 1.21 | 7.41 | 67.984 | ​0.001​ | ​0.001​ |
| HPIV-1 | 1.1 | 1.74 | 0.51 | 12.742 | ​0.002​ | ​0.005​ |
| HPIV-2 | 0.18 | 0.4 | 0.15 | 1.988 | 0.37 | 0.412 |
| HPIV-3 | 0.92 | 0 | 0.21 | 11.291 | ​0.004​ | ​0.009​ |
| HPIV-4 | 1.83 | 0.94 | 0.51 | 11.643 | ​0.003​ | ​0.007​ |
| HCoV-229E | 0 | 0 | 0 | na | na | na |
| HCoV-OC43 | 0 | 0 | 0 | na | na | na |
| HCoV-HKU1 | 0.18 | 0 | 0.03 | na | na | na |
| HCoV-NL63 | 0 | 0 | 0 | na | na | na |
| Bacterial positive rate | 21.25 | 26.98 | 23.17 | 6.744 | ​0.034​ | 0.057 |
| K.pneumoniae | 4.95 | 2.55 | 0.75 | 60.31 | ​0.001​ | ​0.004​ |
| S.pneumoniae | 6.23 | 7.52 | 4.61 | 11.43 | ​0.003​ | ​0.012​ |
| S.aureus | 2.38 | 4.56 | 2.11 | 14.883 | ​0.001​ | ​0.004​ |
| L.pneumophila | 0.37 | 0.54 | 0.39 | 0.344 | 0.842 | 0.936 |
| H.influenzae | 9.16 | 13.02 | 14.97 | 13.867 | ​0.001​ | ​0.004​ |
| P.aeruginosa | 1.28 | 1.74 | 2.71 | 5.682 | 0.058 | 0.081 |
| M.catarrhalis | 0.55 | 1.07 | 0.6 | 2.163 | 0.339 | 0.396 |
| M.pneumoniae | 0.18 | 0 | 0.96 | 10.431 | ​0.005​ | ​0.013​ |
| C.pneumoniae | 0 | 0 | 0.03 | 0.389 | 0.823 | 0.936 |

Note: na stands for no value.

Table S2. The positive rate of pathogens in male and female.

| **Pathogens** | **Male(%)** | **Female(%)** | **X^2^** | **Raw P-value** | **​FDR adjusted P​** |
| --- | --- | --- | --- | --- | --- |
| Overall positive rate | 58.84 | 55.16 | 6.355 | ​0.012​ | ​0.036​ |
| Viral positive rate | 44.12 | 44.53 | 0.079 | 0.778 | 0.847 |
| SARS-CoV-2 | 9.28 | 10.99 | 3.698 | 0.057 | 0.114 |
| RhV | 3.16 | 2.23 | 3.776 | 0.57 | 0.847 |
| IAV | 16.87 | 17.33 | 0.171 | 0.696 | 0.847 |
| IBV | 7.04 | 7.86 | 1.117 | 0.312 | 0.52 |
| RSV | 0.72 | 1.07 | 1.646 | 0.212 | 0.424 |
| HBoV | 0.3 | 0.22 | 0.229 | 0.775 | 0.847 |
| HMPV | 0.55 | 0.45 | 0.24 | 0.68 | 0.847 |
| HAdV | 6.83 | 4.42 | 12.532 | ​0.001​ | ​0.006​ |
| HPIV-1 | 0.72 | 0.85 | 0.257 | 0.62 | 0.847 |
| HPIV-2 | 0.13 | 0.27 | 1.182 | 0.331 | 0.52 |
| HPIV-3 | 0.34 | 0.18 | 1.118 | 0.389 | 0.598 |
| HPIV-4 | 0.76 | 0.71 | 0.031 | 1 | 1 |
| HCoV-NL63 | 0.004 | 0 | na | na | na |
| HCoV-229E | na | na | na | na | na |
| HCoV-OC43 | na | na | na | na | na |
| HCoV-HKU1 | na | na | na | na | na |
| Bacterial positive rate | 26.32 | 20.63 | 20.626 | ​0.001​ | ​0.005​ |
| K.pneumoniae | 1.73 | 1.34 | 1.151 | 0.339 | 0.485 |
| S.pneumoniae | 5.78 | 4.73 | 2.513 | 0.114 | 0.19 |
| S.aureus | 2.61 | 2.46 | 0.117 | 0.779 | 0.779 |
| L.pneumophila | 0.42 | 0.4 | 0.011 | 1 | 1 |
| H.influenzae | 16.2 | 11.61 | 20.129 | ​0.001​ | ​0.005​ |
| P.aeruginosa | 2.61 | 2.14 | 1.097 | 0.334 | 0.485 |
| M.catarrhalis | 0.72 | 0.63 | 0.145 | 0.723 | 0.779 |
| M.pneumoniae | 0.89 | 0.54 | 1.982 | 0.167 | 0.278 |
| C.pneumoniae | 0.04 | 0 | 0.945 | 1 | 1 |

Note: na stands for no value.

Table S3. The positive distribution of respiratory pathogens in different ages group.

| Pathogens | <18 yrs (%) | 18-60 yrs (%) | ≥60 yrs (%) | X^2^ | Raw P-value | FDR adjusted P​ |
| --- | --- | --- | --- | --- | --- | --- |
| Overall positive rate | 62.76 | 54.58 | 63.86 | 27.021 | ​0.001​ | ​0.001​ |
| Viral positive rate | 47.22 | 43.2 | 45.18 | 5.794 | 0.055 | 0.069 |
| SARS-CoV-2 | 3.99 | 11.51 | 27.11 | 109.351 | ​0.001​ | ​0.001​ |
| RhV | 4.16 | 2.28 | 0.6 | 14.572 | ​0.001​ | ​0.002​ |
| IAV | 13.38 | 18.76 | 11.45 | 21.771 | ​0.001​ | ​0.001​ |
| IBV | 7.56 | 7.62 | 3.01 | 4.907 | 0.086 | 0.096 |
| RSV | 1.83 | 0.52 | 1.2 | 17.125 | ​0.001​ | ​0.002​ |
| HBoV | 0.25 | 0.28 | 0 | 0.477 | 0.788 | 0.788 |
| HMPV | 1 | 0.34 | 0 | 8.518 | ​0.014​ | ​0.023​ |
| HAdV | 15.96 | 2.07 | 1.2 | 323.452 | ​0.001​ | ​0.001​ |
| HPIV-1 | 0.5 | 0.89 | 0.6 | 1.847 | 0.397 | 0.441 |
| HPIV-2 | 0.33 | 0.15 | 0 | 1.767 | 0.413 | 0.441 |
| HPIV-3 | 0.25 | 0.28 | 0 | 0.477 | 0.788 | 0.788 |
| HPIV-4 | 0.91 | 0.68 | 0.6 | 0.708 | 0.702 | 0.737 |
| HCoV-229E | 0 | 0 | 0 | na | na | na |
| HCoV-OC43 | 0 | 0 | 0 | na | na | na |
| HCoV-HKU1 | 0 | 0 | 0 | na | na | na |
| HCoV-NL63 | 0.08 | 0 | 0 | na | na | na |
| Bacterial positive rate | 31.26 | 20.33 | 30.72 | 63.027 | ​0.001​ | ​0.001​ |
| K.pneumoniae | 0.5 | 1.85 | 3.01 | 13.044 | ​0.001​ | ​0.003​ |
| S.pneumoniae | 7.23 | 4.5 | 6.02 | 13.263 | ​0.001​ | ​0.003​ |
| S.aureus | 2.33 | 2.59 | 3.01 | 0.404 | 0.817 | 0.817 |
| L.pneumophila | 0.58 | 0.34 | 0.6 | 1.409 | 0.494 | 0.618 |
| H.influenzae | 22.69 | 10.64 | 15.66 | 106.383 | ​0.001​ | ​0.001​ |
| P.aeruginosa | 2.41 | 2.28 | 4.22 | 2.539 | 0.281 | 0.351 |
| M.catarrhalis | 0.67 | 0.49 | 4.22 | 32.774 | ​0.001​ | ​0.001​ |
| M.pneumoniae | 1.33 | 0.52 | 0 | 9.251 | ​0.010​ | ​0.013​ |
| C.pneumoniae | 0.08 | 0 | 0 | 2.833 | 0.243 | 0.304 |

Note: na stands for no value.

Table S4: The co-infection rate of different pathogens at different stage.

| **Pathogens** | **Stage1**  **(%)** | **Stage2**  **(%)** | **Stage3**  **(%)** | **X^2^** | **Raw P-value** | **​FDR adjuste P​** |
| --- | --- | --- | --- | --- | --- | --- |
| HAdV | 33.33 | 33.33 | 37.8 | 0.121 | 0.942 | 0.942 |
| SARS-CoV-2 | na | 32.95 | 27.23 | 1.511 | 0.219 | 0.383 |
| IAV | 27.09 | 30.68 | 23.74 | 2.325 | 0.313 | 0.438 |
| ​IBV​ | ​50.00​ | ​58.33​ | ​26.63​ | 7.641 | ​0.022​ | ​0.077​ |
| H.influenzae | 50 | 60.82 | 52.31 | 2.617 | 0.27 | 0.42 |
| S.pneumoniae | 79.41 | 67.86 | 69.28 | 1.59 | 0.452 | 0.574 |
| S. aureus | 76.92 | 70.59 | 72.86 | 0.194 | 0.908 | 0.942 |

Note: na stands for no value.

Table S5: The co-infection rate of different pathogens at male and female.

| **Pathogens** | **Male(%)** | **Female(%)** | **X^2^** | **Raw P-value** | **​FDR adjusted P​** |
| --- | --- | --- | --- | --- | --- |
| HAdV | 38.89 | 35.35 | 0.194 | 0.659 | 0.769 |
| SARS-CoV-2 | 30 | 30.49 | 0.0001 | 0.989 | 0.989 |
| ​IAV​ | ​29.50 | ​21.13​ | 6.843 | ​0.009​ | ​0.021​ |
| IBV | 28.74 | 27.84 | 0.004 | 0.947 | 0.989 |
| H.influenzae | 53.39 | 53.46 | 0.0001 | 1 | 1 |
| S.pneumoniae | 69.34 | 71.7 | 0.066 | 0.797 | 0.93 |
| S. aureus | 69.35 | 76.36 | 0.411 | 0.521 | 0.769 |

Table S6: The co-infection rate of different pathogens at different age groups.

| Pathogens | <18 yrs (%) | 18-59 yrs (%) | ≥60 yrs (%) | X2 | Raw P value | ​FDR adjusted P​ |
| --- | --- | --- | --- | --- | --- | --- |
| HAdV | 38.02 | 35.82 | 50 | 0.236 | 0.889 | 0.889 |
| SARS-CoV-2​ | 54.17​ | 28.42​ | 22.22​ | 14.908 | 0.001​ | 0.003​ |
| IAV​ | 37.27​ | 22.53​ | 15.79​ | 15.537 | 0.0004​ | 0.002​ |
| IBV | 31.87 | 25.91 | 80 | 7.855 | 0.020​ | 0.047 |
| H.influenzae​ | 60.44​ | 48.70​ | 42.31​ | 9.791 | 0.007​ | 0.016​ |
| S.pneumoniae | 77.01 | 65.75 | 80 | 3.778 | 0.151 | 0.211 |
| S.aureus | 74.07 | 71.08 | 80 | 0.25 | 0.882 | 0.889 |

Table S7: The co-infection rate of different pathogens at different seasons.

| Pathogens | Spring (%) | Summer (%) | Autumn (%) | Winter (%) | X2 | Raw P-value | FDR adjusted P​ |
| --- | --- | --- | --- | --- | --- | --- | --- |
| HAdV | 40 | 33.33 | 44.44 | 37.14 | 0.365 | 0.947 | 0.947 |
| SARS-CoV-2 | 26.05 | 33.65 | 29.27 | 36.79 | 4.511 | 0.211 | 0.369 |
| IAV | 22.32 | 25.61 | 37.23 | 23.21 | 8.642 | ​0.034​ | 0.119 |
| IBV | 21.65 | 41.67 | 48 | 25.54 | 13.429 | ​0.004​ | ​0.028​ |
| H.influenzae | 58.11 | 55.17 | 54.76 | 50.77 | 2.388 | 0.496 | 0.62 |
| S.pneumoniae | 74.58 | 68.97 | 73.81 | 66.67 | 1.346 | 0.718 | 0.838 |
| S.aureus | 81.82 | 73.53 | 66.67 | 70 | 1.686 | 0.64 | 0.747 |
