# Supplementary material for: The dynamic etiology and epidemiological patterns of acute respiratory tract infections during and post non-pharmacological interventions of SARS-CoV-2 in Shenzhen, China: a two years’ prospective cohort study from June 2022
Source: Front Cell Infect Microbiol. 2025 Sep 19;15:1599536. doi: 10.3389/fcimb.2025.1599536 (PMC12491313; doi:10.3389/fcimb.2025.1599536)
Supplement: Supplementary file 7 [file Table2.docx]

Supplementary materials and methods

Table 1. Primers and probers in this study.

| Pathogens | Primer Numeber | Sequence | Fluorescent marker |
| --- | --- | --- | --- |
| SARS-CoV-2 | HKU-N-F | TAATCAGACAAGGAACTGATTA |  |
|  | HKU-N-R | CGAAGGTGTGACTTCCATG |  |
|  | HKU-N-P | GCAAATTGTGCAATTTGCGG | 5'ROX,3'BHQ2 |
| RhV | RhV-F | TGGACAGGGTGTGAAGAGC |  |
|  | RhV-R | GAAACACGGACACCCAAAGTA |  |
|  | RhV-P | TCCTCCGGCCCCTGAATG | 5'CY5,3'BHQ |
| IAV | IFV-A/F | AAGACCAATCCTGTCACCTCTGA |  |
|  | IFV-A/R | CAAAGCGTCTACGCTGCAGTCC |  |
|  | IFV-A/P | TTTGTGTTCACGCTCACCGTGCC | 5'ROX,3'BHQ2 |
| IBV | IFB-F | GTCCATCAAGCTCCAGTTTT |  |
|  | IFB-R | TCTTCTTACAGCTTGCTTGC |  |
|  | IFB-P | AAGYAGGTGGAGAYGGAGGGTCT | 5'VIC,3'BHQ1 |
| HPIV1 | HPIV1-F | AGCAAACGRATATATTATGATGGGA |  |
|  | HPIV1-R | TGGCTATAGATGTTGCAATATTTGA |  |
|  | HPIV1-P | CTCCCACAGTGCCTCAAAGCTTTAACA | 5'FAM,3'BHQ1 |
| HPIV2 | HPIV2-F | AGAATCAATGCTAGRTATGTACTACA |  |
|  | HPIV2-R | ATTTCATGACYTATGAGCTTCAACT |  |
|  | HPIV2-P | CAGAATGCCATCCGCAAGTCAATGGTA | 5'VIC,3'BHQ1 |
| HPIV3 | HPIV3-F | ACTGAYGAAAGATCTGARGCACA |  |
|  | HPIV3-R | ATACATGGGCATTTGGTAATGATGA |  |
|  | HPIV3-P | ACCTGCAAARGCCGCAATAAGAATAGCA | 5'CY5,3'BHQ3 |
| HPIV4 | PIV4-F | CCACATCAATGCAGAATCAYCTTA |  |
|  | PIV4-R | GGTCTATTGCATCAACTTCAATGAT |  |
|  | PIV4-P | ATTGCTGCCAGAGCCCCAGATG | 5'ROX,3'BHQ2 |
| RSV-A/B | RSVAB-F | GGCAAATATGGAAACRTACGTGAA |  |
|  | RSVAB-R | CATATTGTIAGTGATGCIGGRTCAT |  |
|  | RSVAB-P | CTTCACGARGGCTCCACATACACAGC | 5'CY5,3'BHQ3 |
| HBoV | HBoV-F | ACACCWGTAATTATWTCCACTAACCA |  |
|  | HBoV-R | TCTCCAAATGTTTGWGGIAGTTG |  |
|  | HBoV-P | TGGCAATWCTGTWTCTCATGTTCAYGC | 5'FAM,3'BHQ1 |
| HMPV | HMPV-F | TGGGACAAATCATMATGTCTCGYAA |  |
|  | HMPV-R | CTATCWGGCCAACTCCAGTARTT |  |
|  | HMPV-P | TGCAAATATGAAGTRCGGGGCAA | 5'VIC,3'BHQ1 |
| HAdV | ADV-F | CAGGAYGCYTCGGAGTACCT |  |
|  | ADV-R | GCCACIGTGGGRTTYCTAAAYTT |  |
|  | ADV-P | TGGTGCAGTTYGCCCGYGC | 5'ROX,3'BHQ2 |
| HCoV-229E | 229E-F | TGCTAAAACTGAACCTACAGGTTAC |  |
|  | 229E-R | TAACACCATTTGGGAGCTTTTGAT |  |
|  | 229E-P | CCACACTTCAATCAAAAGCTCCCAAATG | 5'CY5,3'BHQ3 |
| HCoV-HKU1 | HKu1-F | TATCGCCTTGCGAATGAATGTG |  |
|  | HKu1-R | TTGCATCACCACTGCTAGTACCAC |  |
|  | HKu1-P | TGTGTGGCGGTTGCTATTATGTTAAGCCTG | 5'ROX,3'BHQ2 |
| HCoV-OC43 | OC43-F | TCAGGGTTACTATATTGAAGGCTCA |  |
|  | OC43-R | TCTACTACGCGATCCTGCACTA |  |
|  | OC43-P | TTCCAGATCTACTTCGCGCACATCC | 5'FAM;3'BHQ1 |
| HCoV-NL63 | NL63-F | AGGACCTTAAATTCAGACAACGTT |  |
|  | NL63-R | GATTACGTTTGCGATTACCAAGACT |  |
|  | NL63-P | TAACAGTTTTAGCACCTTCCTTAGCAACCCAAACA | 5'VIC;3'BHQ1 |
| *K.pneumoniae* | KP-YF | GAATTCAAAACTACCGTCACCCG |  |
|  | KP-YR | TCACGTCGAGGGAATCATGATAGAA |  |
|  | KP-YP | TCATGAGCAGATCACCCGTCTGTTCCA | 5'FAM,3'BHQ1 |
| *S.pneumoniae* | SP-YF | ACTCGTGCGTTTTAATTCCAGCT |  |
|  | SP-YR | CTTACGCAATCTAGCAGATGAAGCA |  |
|  | SP-YP | CTCCCTGTATCAAGCGTTTTCGGCA | 5'VIC,3'BHQ1 |
| *S.aureus* | SA-YF | TGGTTGATACACCTGAAACAAAGCA |  |
|  | SA-YR | TCTTTGACCTTTGTCAAACTCGACT |  |
|  | SA-YP | ATTGGTCCTGAAGCAAGTGCATTTACGA | 5'CY5,3'BHQ3 |
| *L.pneumophila* | LP-YF | ATCAAGGCATAGATGTTAATCCGGA |  |
|  | LP-YR | ATCCGCTTTCTTATTGAATTCAGCA |  |
|  | LP-YP | AGCGCCACTCATAGCGTCTTGCAT | 5'ROX,3'BHQ2 |
| *H.influenzae* | HI-YF | ACGCTTAACTGGTCAATTCACTACA |  |
|  | HI-YR | TTTCACCTGCATAACGCATAGGA |  |
|  | HI-YP | TGGCGGGAACATCAATGATGACAAACCT | 5'FAM,3'BHQ1 |
| *P.aeruginosa* | PA-YF | AACAAGGTCTGGGAACAGGTCTA |  |
|  | PA-YR | TGAAGTGGATGTTGCTGAAGGTCT |  |
|  | PA-YP | CGTTCCGCAGTTCCCACTGCGCGA | 5'VIC,3'BHQ1 |
| *M.catarrhalis* | MC-YF | TGACTTTAAAGCCATGGACGGTAA |  |
|  | MC-YR | TATCAGCAATACTCACAACGCCAT |  |
|  | MC-YP | AGCGTCAGCGGTAACCTAATCTATGC | 5'ROX,3'BHQ2 |
| *M.pneumoniae* | MP-F | ATGTACTATCAGCAAAAGCTCAGTATGG |  |
|  | MP-R | CCACATACCGCTTTAAGTTAGCAA |  |
|  | MP-P | CTAACCAAAACAGCCCTTCAACGGCA | 5'VIC,3'BHQ1 |
| *C.pneumonia* | CP-F | AAGGGCTATAAAGGCGTTGCT |  |
|  | CP-R | TGGTCGCAGACTTTGTTCCA |  |
|  | CP-P | TCCCCTTGCCAACAGACGCTGG | 5'CY5,3'BHQ3 |

**Samples management and laboratory testing(Supplementary)**

The samples were preserved in virus sampling tubes (Virus Sampling Tube by You-Kang Biotech) and then packaged and stored at -80°C on the date. The nucleic acid extraction and pathogen detection were performed on the specimens within 72 hours. For nucleic acid extraction, DNA and RNA of the pathogens were extracted from the samples using a DNA/RNA Extraction Kit (Magnetic Bead Method for DNA/RNA Extraction), following the manufacturer's instructions. The samples were subjected to RT-qPCR using the TransScript Probe Qne-Step qRT-PCR SuperMix kit (Beijing TransGen Biotech). Fluorescent quantitative PCR (qPCR) was performed on the nucleic acids of the samples to detect common respiratory pathogens. The CT value of the pathogens was used to determine whether they were present. A pathogen was considered positive if CT ≤ 37, weakly positive if 37 < CT < 40, and negative if CT ≥ 40. If the CT value was weakly positive, a separate qPCR test was performed on the pathogen and re-well verification was conducted. Positive and negative controls for each relevant pathogen were performed for each test. The reaction mixs is as follows of the RT-qPCR.

Table 2. Componengt of Mix

| Component | Final Concentration | Volumes |
| --- | --- | --- |
| TransScript Probe One-Step RT/R1 Enzyme Mix | - | 2μL |
| 2× PerfecStart Probe One-Step qPCR SuperMix | - | 10μL |
| Primer F（20μM） | 0.4μM | 0.4μL |
| Primer R（20μM） | 0.4μM | 0.4μL |
| Probe（20μM） | 0.4μM | 0.4μL |
| Total RNA | - | 3μL |
| RNase free water | - | add to 20μL |

Table 3. Reaction procedure

| Procedure | Temp | Times | Cycles |
| --- | --- | --- | --- |
| Reverse transcription | 45℃ | 5min | 1 |
| Pre-denaturation | 94℃ | 30s | 1 |
| Denaturation | 94℃ | 5s | 40 |
| Annealing and Extension | 60℃ | 30s |  |

**NPIs in China**

**Timeline and phases of China's NPIs**

**Phase 1:** Emergency Containment (January-March 2020)

Measures: Wuhan city closure on January 23, nationwide travel restrictions, expansion of hospital capacity, centralized isolation of close contacts.

economic disruption.

**Phase 2:** Normalized prevention and control (April-December 2020)

Measure: “External prevention of importation, internal prevention of rebound”, achieved through nucleic acid testing, health codes, and localized outbreak lockdown.

**Phase 3:** Dynamic Zeroing (2021-2022)

Measures: Rapid mass testing, centralized quarantine of asymptomatic infected persons, “closed-loop management” of international travelers.

**Phase 4:** Comprehensive prevention and control (March 2022- December 2022)

Measures: Rapid mass testing, appropriate relaxation of isolation rules (home or centralized isolation), prioritization of vaccination of the elderly, and a shift in focus to reducing severe cases. In December 2022, China reclassified SARS-CoV-2 as a Category B infectious disease to be managed with Category B measures, allowing home isolation and open borders for mild cases, marking a shift toward “living with the virus.”

**Timeline and stage of this study**

**Stage 1:** Jun to Nov 2022 with NPIs. During this phase, which was still in the NPI period, mass screening for SARS-CoV-2 was conducted in a home quarantine or centralized quarantine mode. Consequently, SARS-CoV-2 was not detected in outpatient clinics during this phase.

**Stage 2:** December 2022 to May 2023. This phase is characterized by a model of co-existence with the virus, with no large-scale nucleic acid or enforced quarantine measures. Nevertheless, the population persisted in observing the requisite precautions, including mask-wearing, hand hygiene, and disinfection.During this period, the emergence of SARS-CoV-2 occurred, exhibiting two distinct peaks.

**Stage 3:** Jun 2023 to May 2024. At this stage, a mode of coexistence with the virus was achieved, and society returned to normalcy. Measures such as wearing masks and disinfection were greatly reduced.SARS-CoV-2 achieved coexistence with the virus at this stage, as its virulence continued to weaken, and people's fears and concerns about it gradually diminished.

**Ethics Committee**

This study strictly adhered to the ethical principles of medical research involving human subjects and was formally reviewed and approved by the Ethics Committee of Shenzhen Third People's Hospital (the committee responsible for overseeing the ethical conduct of clinical research within the institution, ensuring compliance with national and international guidelines such as the Declaration of Helsinki). The ethical review focused on safeguarding the rights, safety, and well-being of all participants, including the protection of personal privacy, the minimization of potential risks, and the guarantee of voluntary participation.

Prior to the collection of nasopharyngeal swabs and the gathering of demographic and clinical data, a standardized process of obtaining verbal informed consent was implemented. For adult participants (≥18 years old), trained healthcare personnel clearly explained the purpose of the study, the procedures involved (including sample collection methods and data recording), potential risks (such as mild discomfort during swab collection), benefits (no direct personal benefit, but contributions to public health research on respiratory pathogens), and the right to withdraw from the study at any time without affecting their clinical treatment. Only after confirming that the participants fully understood this information and voluntarily agreed did the team proceed with sample collection.

For pediatric participants (<18 years old), the process was adjusted to respect the special status of minors: verbal informed consent was obtained from their parents or legal guardians, who were provided with the same detailed explanations as adult participants. Additionally, depending on the age and cognitive ability of the child, simplified information about the study (such as explaining that a "soft swab will gently collect a small sample from the nose") was communicated to help them understand and cooperate with the procedure, ensuring that their willingness was also considered to the extent possible.

****Limitations of the Study Design and Population****

An imbalance in sample sizes was observed across the study phases: Stage 1 comprised 11.84% of participants, Stage 2 accounted for 16.16% , and Stage 3 represented the majority at 72.00% . This disparity in sample distribution is largely attributed to the study design: Stage 3 had a longer data collection duration (12 months), whereas Stage 1 and 2 had shorter data collection windows (6 and 7 months ). Furthermore, the more constrained sample sizes in Stages 1 and 2 stemmed primarily from pandemic-related control policies and shortages in medical staff, which contributed significantly to the overall disparity among the three stages. A larger number of cases were collected in Stage 3, attributed to more stable case recruitment during this stage. This larger sample size also facilitates subsequent long-term analyses.

According to negative binomial regression analysis, case numbers exhibited a natural declining trend during stage 1, with a monthly reduction rate of 5% (p=0.012). The policy intervention lift was immediately followed by a 22% short-term decrease in cases (p=0.031); however, this was subsequently offset by a sustained monthly increase of 18% over the longer term (p=0.003). Upon conclusion of the epidemic peak, cases initially fell by 15% (p=0.044), but thereafter entered a phase of markedly accelerated growth, attaining a monthly expansion rate of 24% (p<0.001)(Figure 1 and Table 4).

### Negative Binomial Regression Fit with Monthly Adjustment


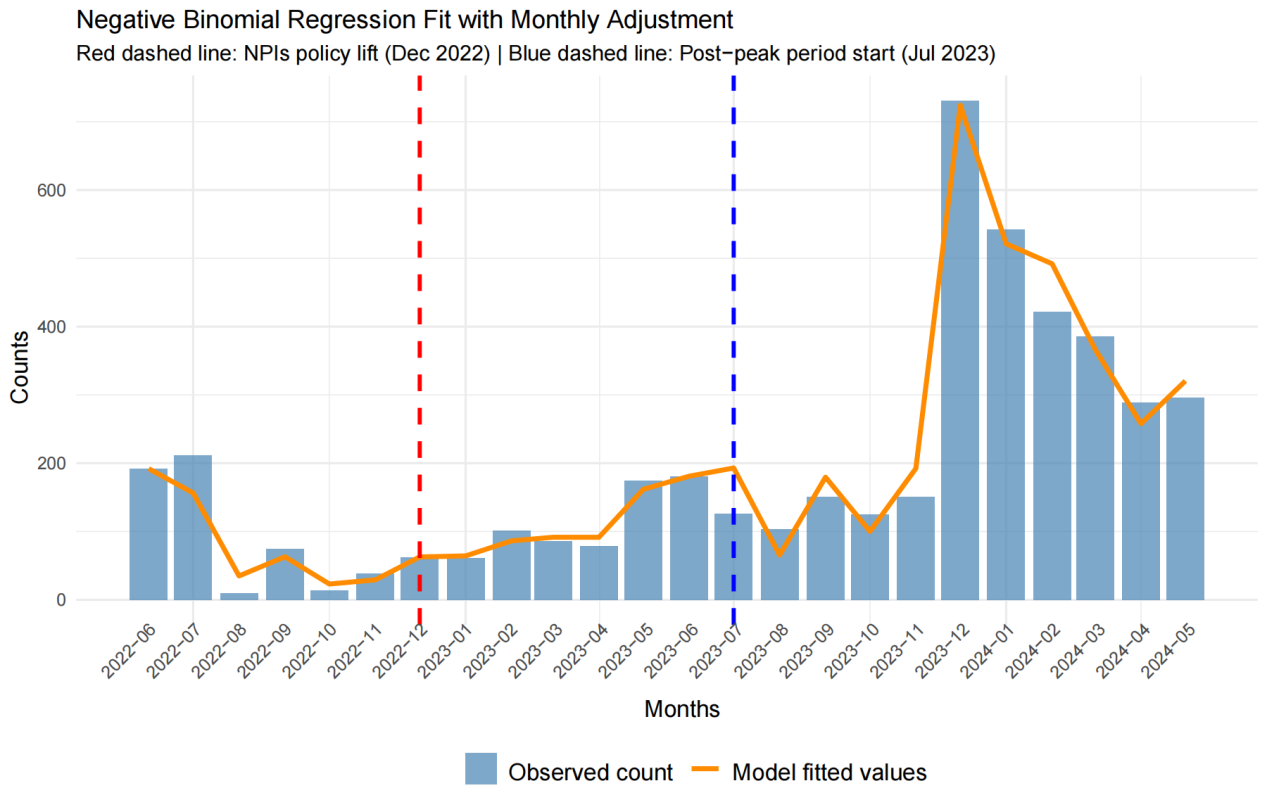
Figure 1. Negative Binomial Regression Fit with Monthly Adjustment in three stages.

Table 4. Key Parameter Estimates of Negative Binomial Regression

| Parameter | IRR | 95%CI | p-value | Interpretation |
| --- | --- | --- | --- | --- |
| **Overall time trend**​ | 0.95 | [0.91-0.99] | 0.012 | Natural monthly decrease of 5% without interventions |
| **Policy lift immediate effect**​ | 0.78 | [0.62-0.98] | 0.031 | 22% immediate reduction after policy change |
| **Policy lift long-term effect**​ | 1.18 | [1.06-1.32] | 0.003 | 18% monthly increase post-intervention |
| **Post-peak immediate effect**​ | 0.85 | [0.73-1.00] | 0.044 | 15% reduction following peak |
| **Post-peak long-term effect**​ | 1.24 | [1.14-1.35] | <0.001 | 24% monthly acceleration in growth |
